# Neglected and Underutilised Crops: A Systematic Review of Their Potential as Food and Herbal Medicinal Crops in South Africa

**DOI:** 10.3389/fphar.2021.809866

**Published:** 2022-01-20

**Authors:** Fhatuwani Nixwell Mudau, Vimbayi Grace Petrova Chimonyo, Albert Thembinkosi Modi, Tafadzwanashe Mabhaudhi

**Affiliations:** ^1^ School of Agricultural, Earth and Environmental Sciences, University of KwaZulu-Natal, Pietermaritzburg, South Africa; ^2^ Centre for Transformative Agricultural and Food Systems, School of Agricultural, Earth and Environmental Sciences, University of KwaZulu-Natal, Pietermaritzburg, South Africa; ^3^ International Maize and Wheat Improvement Center (CIMMYT), Harare, Zimbabwe; ^4^ International Water Management Institute (IWMI-GH), West Africa Office, Kumasi, Ghana

**Keywords:** food and nutrition security (FNS), nutraceutical, orphan crops, pharmaceutical (PhC), sustainable diets

## Abstract

The African continent harbours many native species with nutraceutical and pharmaceutical potential. This study reviewed underutilised crops in South Africa to determine their potential as food and herbal medicinal crops. Over 5,000 species have been identified and earmarked for their medical attributes in formal and informal setups. Researchers, plant breeders and policymakers have mostly ignored the development potential of these crops. Consequently, their value chains are poorly developed. In South Africa, there is a wide range of neglected and underutilised crops, which were historically popular and used by communities; however, over the years, they have lost their status within farming systems and been relegated to the status of neglected and underutilised. Recently, driven by the need to transition to more sustainable and resilient food systems, there has been renewed interest in their potential as food and herbal medicinal crops to establish new value chains that include vulnerable groups. They are now gaining global attention, and their conservation and sustainable utilisation are now being prioritized. The review confirmed that several of these crops possess nutraceutical and pharmaceutical properties, highlighting their potential for development as food and herbal medicines. However, current production levels are too low to meet the requirements for industrial development; research and development should focus on all aspects of their value chain, from crop improvement to utilisation. A transdisciplinary approach involving a wide range of actors is needed to develop the identified neglected and underutilised crops’ potential as food and herbal medicinal crops and support the development of new and inclusive value chains.

## 1 Introduction

Rural communities within South Africa (SA) practise rainfed subsistence agriculture and generally derive low economic returns from farming activities (Beddington et al., 2011). Poverty, unemployment and food and nutrition insecurity are concentrated within these communities (Shackleton et al., 2008). Also, these communities are plagued by the coexistence of undernutrition (i.e., thinness, stunting and underweight) and overnutrition (i.e., overweight and obesity) or diet-related non-communicable diseases (Tsegay et al., 2014; Govender et al., 2016; Thow et al., 2018; Dakora and Belane, 2019). This phenomenon is known as the triple burden of malnutrition (Modjadji and Madiba, 2019). Increasing agricultural activities within these communities has often been viewed as improving food and nutrition security, reducing household poverty, increasing youth employment, and promoting rural development (RSA, 2010). However, due to water scarcity and farmers being located in marginal production environments, and in part changing climate and low capacity to adapt, productivity remains low. It is argued that current agricultural activities within these communities are too mainstream and lack the necessary innovation to allow rural economic development. Given these challenges, there is a need to embrace a new paradigm that promotes context-specific best-bet agricultural technologies that can perform under climate change and meet market demands for sustainable and healthy diets. One strategy could be to diversify cropping systems to include multipurpose food and herbal medicinal plants with nutraceutical, pharmaceutical and economic potential.

Since recorded history, food and herbal medicinal plants have been an integral part of human existence and the traditional African healthcare system. Africa is endowed with huge agrobiodiversity, which herbalists and traditional healers have histrorically used in prescribing medicines for common ailments. Van Wyk (2011) reported that the “African Plant Checklist and Database Project” identified 50,136 angiosperm taxa that occur in sub-Sharan Africa (32,424 taxa in tropical Africa and 22,755 taxa in southern Africa). Africa is estimated to contain between 40 and 45,000 plants with development potential, with more than 5,000 species already used in the formal and informal market as herbal medicinal plants (Van Wyk, 2011; Tasheva and Kosturkova, 2012). Statistics from southern Africa estimates 3,000 species or 13.8% of the flora (Van Wyk and Gericke, 2000), representing 13.5% of the flora is used for herbal medicines. With increasing health awareness and dietary shifts towards healthier foods, there has been an increase in the popularity, production and marketing of functional food crops such as amaranth (*Amaranthus tricolor* L.), bush tea (*Sutherlandia frutescens* L.), honeybush tea (*Cyclopia* Vent.), ginger [*Siphonochilus aethiopicus* (Schweinf.) B.L. Burtt] and mint (*Mentha spp* L.). These crops serve as dual purpose as functional herbal medicinal and food crops or plant-based dietary compounds for therapeutic, nutraceutical and pharmaceutical benefits.

In response, there has been an increase in food and nutrition, health, ethnobiological and ethnopharmacological research along the food-medicine continuum. These research interests have been reinforced by the overwhelming evidence linking diets and health and disease occurrences. However, most studies have primarily addressed popular therapeutic, nutraceutical and pharmaceutical remedies. They have often sidelined traditional and indigenous food crops [hereafter referred to as Neglected and underutilised functional medicinal crop species (NUFMS)]. Furthermore, several attempts to promote these crops have been met with numerous constraints, including but not limited to knowledge about their true medicinal value, poorly developed value chains and conservation practices. It is important to generate information to support the mainstreaming of NUFMS because of their potential to contribute to sustainable rural development, employment creation, food and nutrition security and improved human health and livelihoods. Research, development and innovation have a role to play in commercialising NUFMS through developing high-value products for the food and pharmaceutical industries. The study aimed to identify the range of selected functional neglected and underutilised crops found in South Africa and their nutraceutical and phytochemical properties. In addition, the study outlined a possible strategy for developing the neglected and underutilised functional medicinal crops value chain.

## 2 Materials and Methods

To fulfil the objectives of this research, the methodology of the current study was structured into four phases, namely 1) a general review of key terms and definitions to be used in the review 2) a mixed-methods review of the literature to establish the current status of food and herbal medicinal plants and identify gaps to their mainstreaming, 3) systematic review to quantify the amount of knowledge on a) diversity of functional food and herbal medicinal NUS b), pharmaceutical and nutraceuticals properties, and c) priority NUFMS and 4) proposed production strategy for priority underutilised functional food and herbal medicinal crops. Details of these stages are outlined below.

Phase 1: Definition of terms

Various definitions have been used to describe NUFMS, each with a different meaning and context, causing an incoherent body of literature on these crops. This study identified correct and/or contextualised definitions of key terms in this review. Each definition of a term includes, as a rule, a short formal definition, some additional characteristics and references if available. The terms to be defined include “medicinal plants”, “functional food crops”, “functional medicinal plants”, “Neglected and underutilised crops”, “dietary supplements”, “pharmaceutical”, and “nutraceuticals”. We used official guidelines, position papers, statements and reports of international societies, and original papers and review articles in scientific international journals as references.

Phase 2: Identifying priority functional medicinal crops

This phase of the study was based on the Preferred Reporting Items for Systematic Reviews and Meta-Analyses (PRISMA) statement. The main databases used were SCOPUS and Web of Science. Google Scholar was also used for identifying keywords during the planning of the systematic review.

Phase 3: Status of underutilised functional medicinal crops in South Africa

For this phase, a mixed-method review approach, which included combining quantitative and qualitative research or outcomes with process studies, was used to compile the literature on the status of food and herbal medicinal crops. Where applicable, the emphasis was placed on the use of literature from SA, with some comparisons to regional literature. Narrowing the search to SA allowed for an assessment of local knowledge relative to international knowledge on functional food and herbal medicinal crops.

Phase 4: Identify a production strategy for priority underutilised functional medicinal crops

The NUFMS industry is considered an “uncharted” economy dominated by unsustainable informal production and market systems. Since the development of NUFMS plants in South Africa is at its infancy, a value chain approach was used in developing a guideline for the commercialisation of NUFMS plants.

### 2.1 Search Strategy

A two-step approach was used to focus the review and provide an in-depth assessment of NUFMS nutraceutical and pharmaceutical properties and priorities.

The first stage: planning the review

In this stage, research questions were identified, a protocol was developed, and eventually, the protocol was validated to see if the approach was feasible. The research questions, publication venues, initial search strings, and publication selection criteria were also defined. When all this information was defined, the protocol was revised to see if it represented a proper review protocol.

Research questions:

1) The current review aimed to obtain insight into NUS’s nutraceutical and pharmaceutical properties. Following four research questions (RQs) were defined.
2) What is the body of knowledge around the nutraceutical and pharmaceutical properties of NUFMS?
3) What are the Nutraceutical and pharmaceutical characteristics of NUFMS?
4) Which phytochemicals are associated with the Nutraceutical and Pharmaceutical characteristic of NUFMS?

Identifying keywords

The second step of the planning stage was an internal process to identify keywords, terms, and phrases used in the actual search strings. The review’s objective was copied and pasted into Google scholar, and the top ten articles that appeared were downloaded and reviewed for keywords, terms and phrases. After identifying the articles, we verified the relevance and reliability of each article by searching for them in indexed databases (Scopus and Web of Science).

The ten articles were (Rigat et al., 2009; Dansi et al., 2012; Donno et al., 2015; Bhartiya et al., 2015; Campanaro et al., 2019; Deb et al., 2019; Lambein et al., 2019; Donno and Turrini, 2020; Kumari and Chaudhary, 2020; Udeh et al., 2020)

The second stage: a systematic review

We reviewed the ten articles, and a total of 34 terms (Table 1) were identified for use as keywords. From the review of the ten articles, it was observed that, in many instances, research articles on NUS often insinuate nutraceutical and pharmaceutical benefits without specifically mentioning the actual attribute(s). However, research articles expanded on the phytochemical characteristics that allow nutraceutical and pharmaceutical benefits later in the main text. Therefore, it was important to include *nutraceutical, pharmacological, phytochemical, pharmaceutical and medicinal*. Overall, the main objective was to articulate the nutraceutical and pharmaceutical attributes of NUFMS. Within the ten articles, we identified the following attributes: *antifungal, anti-bacterial, anti-viral, anti-mutagenic, antihepatotoxic, antiinflammatory, antihistaminic, anti-immuno-modulatory, anti-hypolipidemic, anti-diabetic, anti-convulsant, anti-carcinogenic, anti-hypolipidemic, anti-acetylcholinesterase, anti-neuropathic, anti-hypertensive, anti-analgesic, anti-helminths, anti-malaria, lactogenic, aphrodisiaque, diuretic, hepatoprotective, hypotensive, and carminative*. We also observed that these terms had variations, e.g., anti-fungal could be written as “antifungal” or “anti-fungal”. These variants were also included in the search string. In the selected ten articles, it was also observed that several terms had been used to refer to neglected and underutilised crops. Using some of the identified terms and incorporating those obtained from expert knowledge, we came up with the following terms *indigenous, neglected, traditional, orphan, native, underutilis(z)ed, future, medicinal crops*, which were also used as keywords.

**TABLE 1:** Results of neglected functional medicinal crop species from the systematic review. ^1^Crop type -Legume (L), Herb (H), Cereal (C), Cucurbit (Cu), Root and tuber (RT), Tree (T), Shrub (S), Leafy vegetable (LV), Pseudocereal (P) ^2^Growth behaviour - Herb (H), Creeper (Cr), Climber (Cl), Tree (T), Shrub (S), ^3^Plant part—Root (R), Shoot (Sh), Seed (Sd), Stem (St), Flower (Fr), Pod (Pd), Leaves (Lv), Bark (B), Corm (Cm), Tuber (Tu).

Common name	Scientific name	Citation frequency	Country of use	Crop type^a^	Growth behaviour^b^	Parts used^c^	Pharmaceutical and nutraceutical properties	References^d^
Honeybush	*Cyclopia* (Vent.) *spp*	5	South Africa	L	S	Lv	Reduce digestive problems, gives relief for arthritis and to treat diabetes, relaxation and stress relief, colic, hypertension and hypotension, chest ailments, diarrhoea, immune-boosting, blood circulation and blood cleanser, kidney ailments, diabetes, eczema (internally), stomach ailments, constipation, appetite stimulant, breastfeed (stimulate milk in the mother), provide nutrition for the baby and animals when mother’s milk dries up, colds and flu, cosmetics, Contains flavonoids and polyphenols. Has anti-tumour, anti-inflammatory, anti-obesity, anti-oxidant, cardioprotective and anti-diabetic properties	Joubert et al. (2011), Joubert et al. (2008), Okole and Odhav (2004), van Wyk, (2008), Van Wyk and Viljoen (2011)
Tigernut	*Cyperus esculentus* (L.)	2	Benin	L	H	Sd	To treat colon cancer, heart disease, diabetics, obesity, gastrointestinal, Aphrodisiac, Carminative, diuretic, emmenagogue, flatulence, indigestion, diarrhoea, dysentery, excessive thirst, vein expansion, and it is gluten-free. Used as a source of crude fibre, calcium and iron	Dansi et al. (2012)
Ground Bean	*Macrotyloma geocarpum* (Harms) Maréchal and Baudet	2	Benin and Togo	L	H	Sd	Source of crude fat, arginine, amino acids, protein, calcium, potassium, Phosphorus Iron, Zinc, Lysine, Methionine, thiamine, Riboflavin, niacin, Phenylalanine, Histidine, Tryptophane	Bedi and Peter-ikechukwu (2015)
Winged bean	*Psophocarpus tetragonolobus* (L.) D.C.	1	India	L	Cl	Sd	Rich in proteins, oils, vitamins and carbohydrates. Has anti-oxidant, anti-inflammatory, anti-nociceptive, anti-bacterial, anti-fungal, anti-proliferative and cytotoxic activity	Chay et al. (2018)
Sword bean	*Canavalia gladiate* (Jacq.) DC.	1	India	L	Cl	Sd, Pd	treatment of vomiting, abdominal dropsy, kidney-related lumbago, asthma, obesity, stomach-ache, dysentery, coughs, headache, intercostal neuralgia, epilepsy, schizophrenia, inflammatory diseases and swellings. It is rich in carbohydrates, proteins, oils and minerals (K, Mg, Ca, P and S)	Vadivel et al. (2010)
Sunn hemp	*Crotalaria juncea* (L.)	1	India	L	S	F, Sh	The plant is used to purify the blood and is used to treat impetigo and psoriasis	Sangakkara et al. (2003)
Lablab	*Dolichos lablab* (L.) *or Lablab purpureus* (L.) Sweet	1	Kenya	L	Cl	Sd	—	Sennhenn et al. (2017)
Pigeon pea	*Cajanus cajan* (L.) Millsp.	3	Canada, Benin, India	L	S	Lv, St, Sd	It is a source of proteins; fibre, calcium, potassium, magnesium, phosphate	Bedi and Peter-ikechukwu, (2015), Chalwe et al. (2002), Ouma and Jeruto (2010)
Winged bean	*Psophocarpus tetragonolobus* (L.) D.C.	1	Malaysia	L	Cl	Sd	Source of peptides and treatment of ailments caused by microbes	Bhattacharjee et al. (2019)
Bambara groundnut	*Vigna subterranea* (L.) Verdc.	2	Benin, Southern Africa	L	H	Sd	Source of moisture, protein, carbohydrate, energy, crude fibre, calcium, potassium, magnesium, sodium, phosphate, iron, zinc, copper, ascorbic acid, B carotene, Lysine, methionine, thiamine, riboflavin, phenylalanine, histidine, niacin, tryptophan, valine, threonine, leucine, isoleucine, fat, ash, phenolics and flavonoids	Chibarabada et al. (2017), Chibarabada et al. (2017), Olayinka Atoyebi et al. (2017)
Velvet bean	*Mucuna pruriens* (L.) DC. var utilis	5	India	L	Cl	Sd	Treatment of cancer and microbial diseases	Ceballos et al. (2012), Janardhanan (2000), Kaizzi et al. (2004), Ojiem et al. (2007), Pugalenthi et al. (2005)
Grass pea	*Lathyrus sativus* (L.)	1	—	L	Cl	Sd	It possesses many pharmacological effects included anti-oxidant, nervous, anti-diabetic, analgesic, anti-pyretic and cardioprotective effects. The current review discussed the chemical constituents and pharmacological effects of Lathyrus sativus. The oil from the seeds is a powerful and dangerous cathartic (stimulating bowel evacuation). It contains starch, cane sugar, leguminvicilin, legumelin, fixed oil, gum resin, oleoresin, alkaloids, carbohydrates, flavonoids, terpenes, phenols, tannins, vitamin C, riboflavin, carotenoids, beta-carotene, proteins and amino acid	Lambein et al. (2019)
Clusterbean	*Cyamopsis tetragonoloba* (L.) Taub	1	India	L	Cl	Sd	Source of amino acids, ascorbic acid, and Lysine used to treat heart diseases and colon cancer	Rani and Punia (2015)
Broad bean	*Vicia faba* (L.)	1	Spain	L	Cl	Sd	protein, carbohydrates, B group vitamins, and minerals, volute vitamins, folic acid, niacin, and vitamin C, dietary fibre and macro and micronutrient	Manners and van Etten (2018)
African yam bean	*Sphenostylis stenocarpa* (Hochst. ex A.Rich.) Harms	1	Benin	L	—	Sd	Source of B carotene and phenolics. Treatment of cancer	Obidiegwu et al. (2020)
Black gram	*Vigna mungo* (L.) Hepper	2	India	L	H	Sd	The seedpods are diuretic and lithotripic, and the inside of the green pods is rubbed on warts to remove them. Source for lipids, proteins, carbohydrates, amino acids, energy, fibre, ash, lysine, phenolics and leucine. Used for the treatment of cancer and heart diseases	Rathore et al. (2012)
Drumstick	*Moringa oleifera* (L.)	7	Nigeria, India, Mauritius, Spain, South Africa	T	T	Sd, Pd, Lv, R	Almost all tree parts are eaten or used as ingredients in traditional herbal medicines. This especially applies to the leaves and pods, commonly eaten in parts of India and Africa. To date, Moringa oleifera may lead to modest reductions in blood sugar and cholesterol. It may also have antioxidant and anti-inflammatory effects and protect against arsenic toxicity. In addition, it has anti-microbial, anti-oxidant, anti-cancer, cardiovascular, hepatoprotective, anti-ulcer, diuretic, anti-urolithiatic, and anti-helminthic. Its multiple pharmaceutical effects are capitalized as therapeutic remedies for various diseases in the traditional medicinal system. Moringa leaves are an excellent source of calcium, potassium Iron, magnesium, phosphorus, zinc, vitamin A, vitamin B1 (thiamine), B2 (riboflavin), B3 (niacin), B-6 folate and ascorbic acid (vitamin C), oils, fatty acids, micro-macro minerals elements and various phenolics	Gangadhar and Praseetha (2019), Abd El-Razik et al. (2019), Neergheen-Bhujun et al. (2020), Palanisamy et al. (2019), Pande et al. (2018), Singh et al. (2016), Tesfay et al. (2011)
Baobab/African Baobab	*Adansonia digitate* (L.)	3	Benin, Ghana	T	T	Fr, Sd, R, B, St, Lv, F, Sh	To treat diabetes, cancer, diuretics, inflammatory, hypolipidemic, fever and flavonoids. It is a source of Arginine, minerals, calcium, sodium, potassium, magnesium, manganese, iron, zinc, phenolics, Vitamin A, B, C; proteins and carbohydrates	Dansi et al. (2012), De Caluwé et al. (2009), Nyadanu and Lowor (2015)
Breadfruit	*Artocarpus altilis* (Parkinson) Fosberg	2	Benin, india	T	T	—	The sources are fats, carbohydrates, proteins, crude fibre, calcium, sodium, phosphate, magnesium, zinc, manganese, potassium, copper, molybdenum, and vitamins. Assists in treating indigestion, diarrhoea, malaria, vomiting and fever. It can be used externally for wound cleaning	Gulla et al. (2020), Singh et al. (2016)
African fan palm	*Borassus aethiopum* (Mart.)	1	Benin	T	T	Fr, R	The roots may be used to treat stomach parasites, bronchitis, sore throats, and asthma. The leaves are said to be an aphrodisiac, and the sap is reported to have many uses. The African fan plant is a good source of protein, fat, ash, fibre, amino acids, aspartic acid, threonine, serine, glutamic acid, proline glycine, methionine, isoleucine, leucine, minerals, sulphur, potassium, magnesium, calcium, sodium, zinc, iron, manganese, and copper	Bolade and Bello (2006)
Blackberry	*Rubus fruticosus* (L.)	1	Benin	T	T	—	Useful in treating cancer, diarrhoea, dysentery, whooping cough, anaemia, toothache, mouth ulcer, sore throat, haemorrhoids, and minor bleeding. Virtually all parts of the plants are used traditionally to treat skin-related diseases, diabetes, diarrhoea, hypertension, cough and bronchitis. Source of protein, crude fibre, ash, carbohydrates, carotenoids, vitamin C, and minerals. Blackberry is also a good source of nutrients; manganese, copper, dietary fibre carbohydrates, zinc, magnesium, minerals, and vitamins	Dansi et al. (2012), Odebunmi and Oluwaniyi (2010)
Mulberry	*Morus alba* (L.)	2	India	T	T	Fr, Lv	Used for treating dizziness, insomnia, premature ageing, skin irritation and DM2. Contains vitamin C, zinc, calcium, iron, potassium, phosphorus, and magnesium	Zhang et al. (2018)
Physic nut	*Jatropha curcas* (L.)	1	India	T	T	Fr, Sd, R, B, St, Lv, F, Sh	Source of Ascorbic acid, phenolics, anthocyanins and flavonoids. Treatment of cancer, inflammation, and diabetes	Achten et al. (2010)
Jackfruit	*Artocarpus heterophyllus* (Lam.)	1	India	T	S	—	Presence of many secondary metabolites, including diterpenoids, sesquiterpenoids, alkaloids, flavonoids, phenols, lignans, coumarins and cyclic peptides. pharmacological activities, such as anti-inflammatory, anti-oxidant, anti-microbial, anti-viral, anti-cancer, anti-diabetic, anti-coagulant, hepatoprotective, analgesic and abortifacient effects	Kushwaha et al. (2021)
Velvet tamarind	*Dialium guineense* (Willd.)	1	Benin	T	H	—	Sources of proteins, carbohydrates and amino acids	Fandohan et al. (2010)
Marvel of Peru or four o’clock flower	*Mirabilis jalapa* (L.)	1	India	S	—	Lv, Sd, Sh, St	Treating ulcers, diarrhoea, boils, stomach-ache, boils, skin diseases and asthma. It is an anti-syphilitic, anti-bacterial and a vermifuge	Bhattacharjee et al. (2019)
Chocolate weed	*Melochia corchorifolia* (L.)	1	India	S	H	Sd, Lv, St	diuretic, purgative, and for vulnerary, aphrodisiac, reduce inflammation, For candida, chagas disease, colic, constipation, contusions, diarrhoea, dysentery, earache, oedema, eczema, freckles, herpes, hives, itch, intestinal parasites, liver problems, pain, skin problems, skin infections, syphilis, vaginal discharge, urinary insufficiency, wounds, worms	Bhattacharjee et al. (2019)
Cannabis	*Cannabis sativa* (L.)	2	Canada	S	S	Sd, L	Cannabis is commonly used for long-term or severe pain, nausea caused by chemotherapy and painful muscle spasms. Cannabis is a good source of essential fatty acids, amino acids, dietary fibre, enzymes, vitamins, proteins, carbohydrates, water, fat, trace amounts of calcium, potassium, sodium, minerals, flavonoids, carotenoids, terpenes and phytocannabinoid acids	Aderinola et al. (2019), Caplan et al. (2019)
Hibiscus or Roselle	*Hibiscus sabdariffa* (L.)	3	Egypt, India, Japan	S	H	Lv, F, Sd	Reduces inflammation treatment of pain, spasms, asthma, insomnia, depression, and loss of appetite	Al-Sayed et al. (2020), Bhattacharjee et al. (2019), Abd El-Razik et al. (2019)
Cape periwinkle; graveyard plant	*Catharanthus roseus* (L.) G.Don	1	Israel	S	H	Lv, Sh	Source of K, Mg, Mn, Fe, CHO, Zn, P and vitamin C. Used for the treatment of diabetes, indigestion, blood pressure. lt also has biotic functions	Levy et al. (1983)
Cassava	*Manihot esculenta* (Crantz)	1	Philippines	S	S	R, Tu, Lv, Sh	Treatment of high blood pressure, liver diseases and fevers, mild laxative, anti-bacterial. Rich in phytochemicals like polyphenols, especially anthocyanins, polysaccharides and organic acids. Source of manganese, copper, molybdenum and ascorbic acid	Publishers (2018)
Donkey berry	*Grewia flavescens* (Juss)	2	Niger, India	S	S	B, Lv, Fr	Can be used for wound cleaning, menstrual, stomach problems. Good source of potassium, calcium, manganese, iron, copper, zinc, crude fibre, ash, and carbohydrates	Gebauer et al. (2013), Kumar et al. (2017)
Carob	*Ceratonia silique* (L.)	2	South Africa	S	S	St, Lv, F, Pd, R	Treatment of Aphrodisiac, diarrhoea, cancer, heart disease, inflammation, microbial. Provide flavonoids, tannins, and steroids	Batlle and Tous (1997), Kleynhans et al. (2018)
Cancer bush	*Sutherlandia frutescens* (L.) R.Br. *or Lessertia frutescens* (L.) Goldblatt and J.C.Manning	3	South Africa	S	S	St, Lv, F, Pd, R	Used to treat chickenpox, diabetes, cancer, menopausal symptoms, influenza, rheumatoid arthritis, peptic ulcers, anxiety, clinical depression, HIV infection and external wounds. Caner bush is amino acids, proline, and alanine	Kleynhans et al. (2018), Masenya et al. (2020), Van Wyk (2011)
Ethiopian eggplant	*Solanum aethiopicum* (L.)	1	Ghana	S	S	Fr	Treatment of cancer and diabetics	Nyadanu and Lowor (2015)
African eggplant	*Solanum macrocarpon* (L.)	2	Ghana	S	S	—	Source of fats, carbohydrates, proteins, crude fibre, Ca, Na, P, Mg, Mn, K, Fe, Zn, Cu, Mo, vitamin A, Vitamin C. Treatment of diabetes, dysentery haemorrhoids, bowel movement and Blood pressure	Nyadanu and Lowor (2015)
Bitter eggplant	*Solatium insanum,* (L.)	1	Sri Lanka	S	S	Sd, Lv, R	Source of fats, CHO, proteins, C-fibre, Ca, Na, P, Mg, Mn, K, Fe, Zn, Cu, Mo and vitamin c	Samarakoon et al. (2018)
Miracle fruit	*Synsepalum dulcificum* (Schumach. and Thonn.) William Freeman Danielferl	1	Africa	S	H	Fr	Source of carbohydrates, vitamin A, vitamin C and phenolics	Tchokponhoué et al. (2017)
Cactus pear	*Opuntia robusta* J.C.Wendl. ex Pfeiff	2	South Africa	S	S	Fr	Used for type 2 diabetes, high cholesterol, obesity, alcohol hangover, colitis, diarrhoea, and benign prostatic hypertrophy (BPH). Good source of minerals, amino acids, vitamin C, E, K, and beta-carotene, flavonoids, and antioxidants	Venter et al. (2019)
Pea eggplant	*Solanum torvum* (Sw.)	2	India	S	S	R, Fr	Source of vitamin C, Leucine, flavonoids and anthocyanin. Treatment of diabetes and gastrointestinal conditions	Bhattacharjee et al. (2019), Nyadanu and Lowor (2015)
Quinoa	*Chenopodium quinoa* C.L. Willdenow (Willd.)	1	China	P	H	Sd	Rich in fibre, minerals, antioxidants, and all nine essential amino acids. Quinoa is a good source of protein, lipids, ash, crude fibre, carbohydrate, and energy	Amjad et al. (2015)
Buck wheat	*Fagopyrum esculentum* (Moench)	3		P	H	Sd, F	Treatment of bacterial diseases	Bashir et al. (2021), Horbowicz and Obendorf (2005), Kumari and Chaudhary (2020)
Amaranth	*Amaranthus spinosus* (L.)	3	India	P	H	Lv, Sh	Amaranth is used to treat diarrhoea, ulcers, swollen mouth and throat. The leafy vegetable is rich in fibre, protein, calories, protein, carbohydrates, fat, manganese, magnesium, phosphorus, iron, selenium, copper	Bhattacharjee et al. (2019), Rastogi and Shukla (2013), Verma et al. (2017)
Amaranth	*Amaranthus tricolor* (L.)	3	India	P	H	Lv, Sh	Treatment of eczema. It is a source of flavonoids, steroids, lipids, carbohydrates, crude fibre, amino acids, minerals, protein, ash, B carotene and phenolics	Bhattacharjee et al. (2019), Rastogi and Shukla (2013), Verma et al. (2017)
Elephant foot yam	*Amorphophalus campanulatus* (Dennst.) Nicolson	1	India	RT	H	Tu	Source of crude fat, crude protein, crude fibre, Calcium, Manganese, iron, zinc, copper, Vitamin A, B-carotene, foliate, lysine and methionine. Used as a diuretic	Bhattacharjee et al. (2019)
Up yam	*Dioscorea bulbiferaa* (L.)	1	India	RT	H	Tu	Source of calcium, manganese, phosphate, iron, zinc, copper, Vitamin A, B-carotene and foliate. Used for the treatment of eczema and inflammation	Bhattacharjee et al. (2019)
Lesser yam	*Dioscorea esculenta* (Lour.) Burkill	2	India, Philippines	RT	H	Tu, St	Lesser yam is used to treat piles, dysentery, syphilis, ulcers, leprosy, diabetes, asthma, cough, and cancer. Nutritional composition includes protein, crude fibre, ash, and fat	Bhattacharjee et al. (2019)
Taro	*Colocasia esculenta* (L.) Schott	5	India, Benin, Philippines, Tanzania	RT	H	Cm, Lv, Sh, St	Treatment of diarrhoea, help control blood sugar, reduce risk of heart disease, weight loss, anti-cancer properties. Good source of protein, carbohydrates, fat, fibre, vitamins, potassium, folate, calcium, magnesium, phosphorus, and iron	Bhattacharjee et al. (2019), Grimaldi et al. (2018), Gulla et al. (2020), Okole and Odhav (2004), Singh et al. (2016), Son et al. (2021)
Greater yam	*Dioscorea alata* (L.)	2	India, Philippines, Sub Saharan Africa	RT	H	Tu	Treatment of ulcers, boost brain health, reduce inflammation, and improve blood sugar control. Source of carbohydrate, vitamin B6, copper, manganese, potassium	Bhattacharjee et al. (2019), Crop and Society (2019), Obidiegwu et al. (2020), Osei et al. (2019), Singh et al. (2016)
Yam	*Dioscorea dumetorum* (Kunth) Pax	4	Benin, India	RT	Cl	Tu	Rich in Vitamin C as well as starch. It contains calcium, phosphorous, thiamine, riboflavin, niacin, oxalic acid, calcium oxalate, sapotoxin and flavones, apigenin and luteolin. Has Anti-microbial, Anti-hepatotoxic, Anti-cancer, Anti-Lipidperoxidative, Anti-bacterial and Anti-fungal, Anti-diabetic, Anti-melanogenic, Anthelmintic, Anti-microbial, Anti-hepatotoxic, Anti-cancer, Anti-Lipidperoxidative, anti-bacterial and Anti-fungal, Anti-diabetic, Anti-melanogenic, Anthelmintic properties	Dansi et al. (2012), Obidiegwu et al. (2020), Paul et al. (2020)
Sweet potato	*Ipomea batatas* (L.) Lam.	3	Benin, Pakistan	RT	Cr	—	Sweet potato may be used to promote gut health, treat cancer, vision, support immune system and brain function. Great source of fibre, minerals and vitamins	Dansi et al. (2012), Galvao et al. (2021), Singh et al. (2016)
Giant taro	*Alocasia macrorrhiza* (L.) G.Don	1	Philippines	RT	H	St, Lv	Provides moisture, crude fat, protein, ash, crude fibre, minerals, calcium, sodium, potassium, magnesium, manganese, phosphate, iron, zinc, copper and phenolics. It treats cancer, heart disease, diabetes, dysentery, inflammation, gonorrhoea, haemorrhages, hypertension, helminths. It is a source of tannins and flavonoids	Publishers (2018)
Ethiopian potato	*Plectranthus edulis* (Vatke) A.J.Paton	1	Ethiopia	RT	H	Tu	Used to treat urinary disorders, including bladder infection (cystitis), prostatic hyperplasia, prostate cancer, lung diseases, and other cancer. Contains fat, fibre, sodium, potassium, carbohydrates, vitamins, minerals and other essential nutrients	Gulla et al. (2020)
Wild ginger	*Siphonochilus aethiopicus* (Schweinf.) B.L.Burtt	3	South Africa	RT	H	—	Used to treat intestinal ailments, relieve stomach aches and cramps. Reduces stress, pain, anxiety. Contains fat, sodium, carbohydrates, sugars, protein, and calories	Geldenhuys, (2007), Okole and Odhav (2004), Xego et al. (2016)
Tannia	*Xanthosoma sagittifolium* (L.) Schott	4	India, Ghana, Philippines	RT	H	Cm, St, Lv, Tu, R	Source of moisture, fats, crude protein, protein, crude fibre, minerals, sodium, potassium, manganese, phosphates, zinc, iron, copper, lysine, methionine, histidine, and Isoleucine	Bhattacharjee et al. (2019), Grimaldi et al. (2018), Nyadanu and Lowor (2015), Singh et al. (2016)
White seed melon	*Cucumeropsis manni* (Naudin)	1	Benin	Cu	Cr	Sd, Fr	Juice from the fruit is mixed with other ingredients to treat the cord-relic of newborn babies until it drops off. Source of carbohydrates, proteins, essential amino acids, fatty acids, minerals, and vitamins	Onawola and Asagbra (2012)
Watermelon	*Citrullus lanatus* (Thunb.) Matsum. and Nakai	1	Benin	Cu	Cr	Sd, Fr	Used to treat urinary tract infection, alcohol poisoning, hypertension, diabetes, gonorrhoea, and diarrhoea. Good source of copper, vitamin B5, lycopene, and vitamin C	Dansi et al. (2012), Gullino et al. (2020)
Bottle gourd	*Lagenaria siceraria* (Molina) Standl.	1	China	Cu	Cr	—	It is a source of crude fats, moisture, proteins, carbohydrates, energy, amino acids, minerals and Vitamin A	Zhang et al. (2020)
Bitter gourd	*Momordica charantia* (L.)	2	India, South Africa	Cu	Cr	Fr	Provides proteins, potassium, iron and fibre. It is used in the treatment of cancer and as an aphrodisiac	Bhattacharjee et al. (2019), Kutu and Magongwa (2017)
Wax gourd	*Benincasa hispida* ((Thunb.) Cogn.)	1	India	Cu	Cr	—	Source of proteins, fibre, amino acids, ascorbic acid and B carotene	Bhattacharjee et al. (2019)
Bitter melon	*Momordica charantia* (L.)	1	Korea	Cu	Cr	Fr	Used to fight cancer, diabetes, and many infectious diseases and treat eye-related diseases. Contains calories, fat, sodium, carbohydrates, fibre, sugar, and protein	Chung et al. (2016)
Pumpkin	*Cucurbita pepo var. styriaca* (L.)	1	Iran	Cu	Cr	Sd, R, F	Source of vitamin A, B carotene, Fat, Protein, Crude fibre, Minerals, Calcium, sodium, potassium, magnesium, phosphates, iron, copper. Assists with bowel movement and treatment of helminths, liver complaints and intestinal diseases	Soltani et al. (2016)
Napier grass	*Pennisetum purpureum* (Schumach)	1	Africa	C	H	—	Treatment of cancer and viral diseases. Reduces inflammation and	Khan et al. (2010)
Pearl millet	*Cenchrus americanus* (L.) Morrone	6	Benin, Kenya, India	C	H	Sd	Treat iron deficiency anaemia, reduce blood sugar levels, aids in weight loss and microbial actions. Good source of energy, moisture, protein, fat, mineral, fibre, carbohydrate, calcium, phosphorus and iron. Control blood sugar, improve digestive health. Good source of vitamins, phosphorus, potassium, antioxidants, niacin, calcium, and iron	Avashthi et al. (2018), Dansi et al. (2012), Gulla et al. (2020), Kamble et al. (2019), Ndiku et al. (2016), Srivastava et al. (2021)
Barley	*Hordeum vulgare* (L.)	1	Himalaya	C	H	Sd	Sources of Na, P, Mg, Mn, K, Fe, Zn, Cu, Mo, Ca, and oleic, palmitic and stearic acids	Bungla et al. (2017)
Proso millet	*Panicum miliaceum* (L.)	1	Himalaya	C	H	Sd	Source of moisture, fat, protein, ash, carbohydrate, amino acid and Vitamin A	Bungla et al. (2017)
Fonio millet	*Digitaria exilis* (Kippist) Stapf	1	Benin	C	H	—	The amino acid methionine is important for the body’s cartilage production. Helps strengthen nails and hair. Fonio millet is a good source of thiamine niacin, riboflavin carbohydrates, protein, fat, fibre, and iron	Dansi et al. (2012)
Foxtail millet	*Setaria italica* (L.) P. Beauvois	1	Himalaya	C	H	Sd	Good for cardiac health, regulates blood sugar level, lower blood cholesterol, improves digestion and immunity	Bungla et al. (2017)
Finger millet	*Eleusine coracana* (L.)	4	India, United States, Canada	C	H	Sd, Lv	Source of protein, carbohydrates, crude fibre, energy, thiamine, riboflavin, niacin, phenylalanine, threonine, valine, leucine and isoleucine	Bungla et al. (2017); Hall et al. (2021); Kamble et al. (2019); Singh et al. (2010)
Sorghum	*Sorghum bicolor* (L.)	2	Benin	C	H	—	Source of moisture, fat, protein, ash, carbohydrate, amino acid and Vitamin A, Source of phenolics	—
Maize	*Zea mays* (L.)	4	Philippines, Iran, Vietnam	C	H	Sh, Sd	Source of moisture, fat, protein, ash, carbohydrate, amino	Ndiku et al. (2016), Sales et al. (2018), Sarkar et al. (2020), Sharifi-Rad et al. (2016)
Scarlet pimpernel	*Chenopodium album* (L.)	1	Egypt	H	H	—	Source of phenolics, Treat iron deficiency anaemia, reduce blood sugar levels, and microbial actions	Abd El-Razik et al. (2019)
Bush tea	*Athrixia phylicoides* DC.	5	South Africa	H	S	Lv	cleansing or purifying the blood, treating boils, headaches, infected wounds, cuts, and the solution may also be used as a foam bath. Treatment of various ailments such as boils, acne, colds, loss of voice, and throat infection as a gargle. significantly high polyphenols, tannins, antioxidants, quercetin, flavonoids, alkaloids, polysaccharides, amino acids, lipids, vitamins, and inorganic elements	De Caluwé et al. (2009), Okole and Odhav, (2004), Slabbert et al. (2019), Zonyane et al. (2019)
Sweet clover	*Melilotus officinalis* (L.) Pall	1	Egypt	H	H	—	Used to treat microbial infections and diabetes. It is a source of carbohydrates, sodium, potassium, magnesium, manganese, phosphates, iron, zinc, Vitamin A	Abd El-Razik et al. (2019)
Lemongrass	*Cymbopogon flexuosus* (Nees ex Steud.) W.Watson	2	India, Saudi Arabia	H	H	Fr	Used to treat stomach and intestinal spasms, ache, high blood pressure, convulsion, pain, vomiting, cough, achy joint, and fever. Rich in minerals, various essential nutrients and vitamins	Dagar et al. (2013), Sujatha et al. (2011)
Rapeseed/Sarson	*Brassica napus* (L.)	2	United States, India	H	H	—	Source of moisture, fat, CHO, protein, fibre, Fe, Zn, B carotene, lysine, phenolics and flavonoids	Saroop and Kaul, (2015), Velasco et al. (2007)
Water hyssop	*Bacopa monnieri* (L.) Pennell	2	India	H	H	Lv	Contains powerful antioxidants, may reduce inflammation, boost brain function, reduce ADHD symptoms, may prevent anxiety and stress. Source of carbohydrates, fat, proteins, and minerals	Bhattacharjee et al. (2019), Verma et al. (2017)
Safflower	*Carthamus tinctorius* (L.)	2	China, Pakistan	H	H	Sd, F	Used to traditionally treat painful joints, trauma, dysmenorrhea, amenorrhea, postpartum, and abdominal pain. The nutritional value includes copper, tryptophan, fat, vitamin B1, and phosphorus	Koley et al. (2020), Liu et al. (2016)
Fennel flower	*Nigella sativa* (L.)	1	Iran, Egypt	H	H	R, Sh	Treatment of diabetes and gastro-intestinal bowel movement. It is used as an aphrodisiac, diuretic and assists with indigestion	Al-Sayed et al. (2020), Abd El-Razik et al. (2019), Srivastava et al. (2021)
Ladies’ fingers or Okra	*Abelmoschus esculentus* (L.) Moench	1	Africa, India, Iran	H	H	Sd, R, St, Fr	An infusion of the root is used to treat syphilis, the juice of the roots is used to treat cuts, wounds, and boils. Good source of calories fats, sodium, potassium, carbohydrates, protein, dietary fibre, proteins, vitamins, and iron	Okole and Odhav (2004), Singh et al. (2016), Xavier et al. (2019)
Plantain	*Plantago major* (L.)	3	Sweden, Egypt	H	H	Lv	Helps with memory. Sources of carbohydrates. fibre, Ca, K, P, Fe, Cu, Vitamin A	Abd El-Razik et al. (2019), Paul et al. (2020), Zubair et al. (2011)
Toothache plant	*Acmella oleracea* (L.) R.K.Jansen	1	Benin	H	H	—	Used to treat toothache, throat and gum infections, stomach, diuretic, dry mouth, and gastric ulcers. Good source of fat, carbohydrates, nitric oxide, and hydroxytoluene	Dansi et al. (2012)
Creeping woodsorrel	*Oxalis corniculata* (L.)	1	India	H	H	Lv	Source of proteins, moisture, carbohydrates, minerals, Ca, Mg, Na, Mn, P, Fe, vitamin A, Vitamin C, B carotene, oleic and palmitic acid. Treatment of heart disease, cancer, inflammation, blood pressure, hypertension, ulcers, tannins and flavonoids	Bhattacharjee et al. (2019)
Chinese water chestnut	*Eleocharis dulcis* (Burm.f.) Trin. ex Hensch	1	India	H	H	Tu	Source of fats and lipids, crude proteins, energy, crude fibre, calcium, manganese, sodium, potassium, iron, zinc, copper and vitamin A and C. It is used for the treatment of gastrointestinal problems, including dysentery. It is used as a diuretic and treatment of gonorrhoea and fever	Bhattacharjee et al. (2019)
Lamb’s quarters	*Chenopodium album* (L.)	3	India, Egypt	LV	H	Lv, Sh	Source of flavonoids. Treatment of inflammation, cardiovascular diseases, diarrhoea and helminths	Bhattacharjee et al. (2019), Abd El-Razik et al. (2019), Verma et al. (2017)
False sesame	*Ceratotheca sesamoides* (Endl.)	1	Benin	LV	H	Lv	Source of proteins, carbohydrates and amino acids	—
Black sesame	*Sesamum radiatum* (Schumach. and Thonn)	1	Benin	LV	H	Sd, Lv	Sources of carbohydrates. fibre, Ca, K, P, Fe, Cu, Vitamin A, Vitamin C, ascorbic acid and B carotene	Dansi et al. (2012)
—	*Crassocephalum rubens* (Juss. and Jacq.) S	2	Benin	LV	H	Lv	Used to treat indigestion, upset stomach, headaches, epilepsy, fresh wounds, to stop nose bleeding, swollen lips and sleeping sickness. Good source of crude protein, lipid, ash, fibre, carbohydrates, and energy	Dansi et al. (2012), Nyadanu and Lowor (2015)
Spiderflower	*Cleome gynandra* (L.)	3	Benin, India	LV	H	Lv	Source of phenolics. Treatment of cancer, viral diseases, diabetes, hypertension, inflammatory conditions, ulcers and wound cleaning	Dansi et al. (2012), Okole and Odhav (2004), Saroop and Kaul (2015)
Jute mallow	*Corchorus olitorius* (L.)	2	Benin, Ghana	LV	H	Lv, Sd	A good remedy for aches, pains, dysentery, enteritis, fever, pectoral pains, ascites, piles, and tumours. It is also a rich source of potassium, iron copper, manganese and zinc	Dansi et al. (2012), Nyadanu and Lowor (2015)
Thickhead, redflower ragleaf	*Crassocephalum crepidioides* (Benth.) S.Moore	2	Benin, Ghana	LV	H	Lv	Source of proteins, iron and potassium	Dansi et al. (2012), Nyadanu and Lowor (2015)
Wild mustard	*Brassica juncea* (L.)	1	India	LV	H	—	Source of moisture, fats, proteins, carbohydrates, fibre, minerals, Ca, Na, K, Mg, Mn, P, linoleic, oleic, Palmitic and stearic acid	Gangadhar and Praseetha (2019)
Kales	*Brassica oleracea* (L.)	4	Spain, Netherlands	LV	H	Lv	Source of proteins, lipids Cu, Fe, Zn, Na, Zn, Mg, Mn and vitamin c. Used for treating diabetes, inflammation, hypertension, Malaria, liver complaints, helminths, hepatic insufficiency and has biotic functions	Giambanelli et al. (2016), Nyadanu and Lowor, (2015), Sharifi-Rad et al. (2016), Velasco et al. (2007)
Common dandelion	*Taraxacum officinale* F. H. Wigg	1	Iran	LV	H	R, Sh	Source of moisture, fat, protein, carbohydrates, crude fibre, energy, calcium, potassium, iron, ascorbic acid, phosphorus, copper. Used in treating inflammation, blood pressure, lactogenic cancer, viral diseases and haemorrhoids. It is used as an aphrodisiac	Sharifi-Rad et al. (2016)
Purslane	*Portulaca oleracea* (L.)	1	NA	LV	H	Sd	Can be used as a febrifuge, anti-septic, vermifuge. Has a good content in sodium, potassium, carbohydrates, protein, vitamin C	Srivastava et al. (2021)
Hyacinthus	*Hyacinthaceae*	2	South Africa	LV	Cr	R, Fr, Sh	Used to treat rheumatism, cardiac, urinary infection, dermatological problems, stomach, haemorrhoid, and prostate disease. Hyacinthus is a good source of crude lipids, ash, fibre, proteins and minerals; potassium, and sodium	Masondo et al. (2014), McCartan and Van Staden (1999)

a Crop type -Legume (L), Herb (H), Cereal (C), Cucurbit (Cu), Root and tuber (RT), Tree (T), Shrub (S), Leafy vegetable (LV), Pseudocereal (P).

b Growth behavior—Herb (H), Creeper (Cr), Climber (Cl), Tree (T), Shrub (S).

c Plant part—Root (R), Shoot (Sh), Seed (Sd), Stem (St), Flower (Fr), Pod (Pd), Leaves (Lv), Bark (B), Corm (Cm), Tuber (Tu).

d Reference cites can be found in Supplementary information document 1

To address RQ 1) and 2), searches were conducted using above stated keywords and search combinations outlined in Table 1.

### 2.2 Data Mining, Analysis and Presentation

**FIGURE 1:**
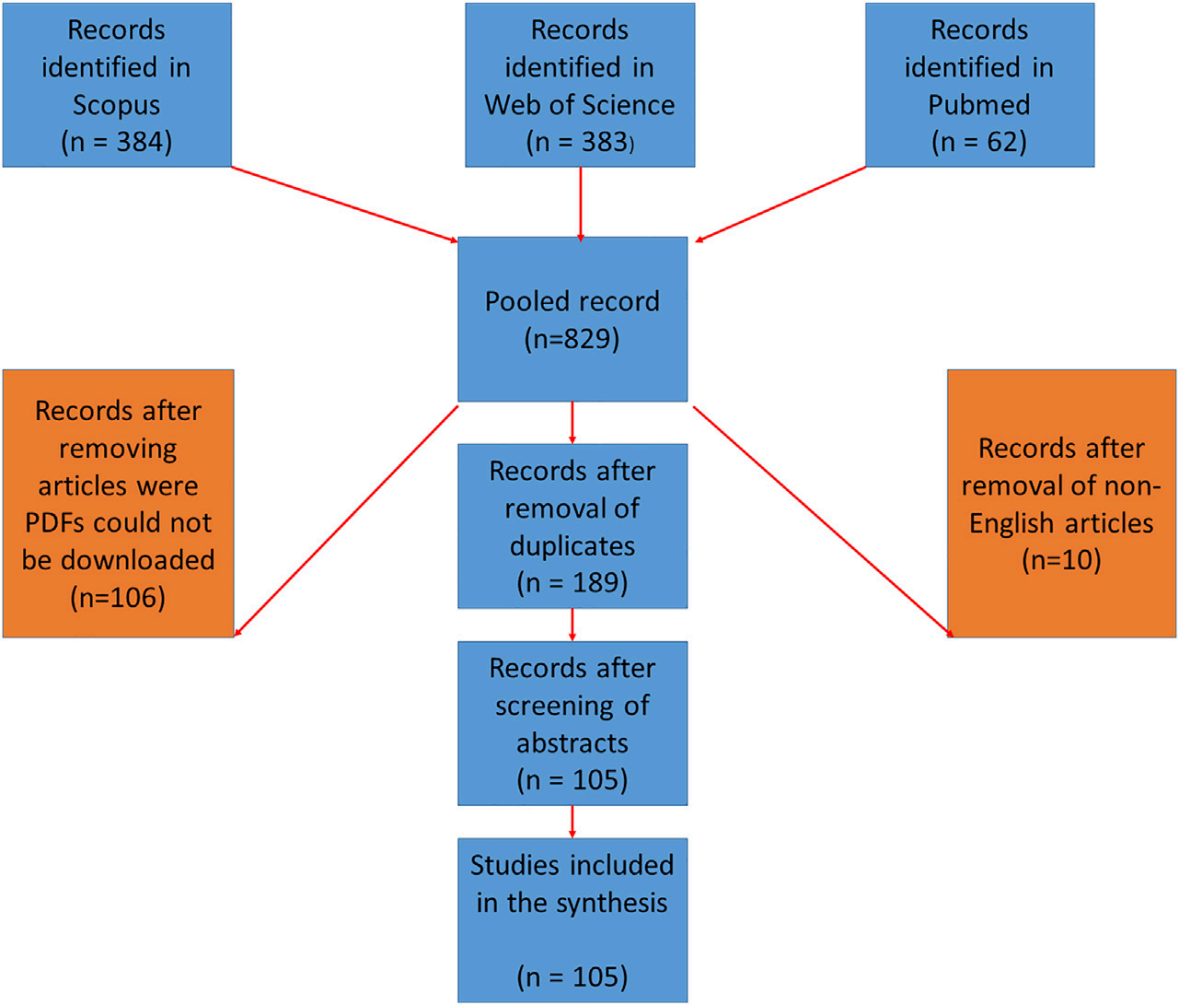
PRISMA diagram of the selected articles for the literature synthesis.

The second stage was conducting the review to identify priority NUFMS. When conducting the literature search, the cluster of key terms was systematically used in each database, and these were later combined to come up with a list of articles. The lists from the different databases were combined and duplicates removed (Supplementary Table S1 and Figure 1). The list of articles was exported to Excel, where data on authors, year of publication, type of publication, crop species and type, nutraceutical and pharmacological characteristics, phytochemical properties and any other information to help answer the research questions were extracted and stored. Articles excluded from the final list included those discussing crop species not found in Africa, whose focus was very broad and not on nutraceutical and pharmacological properties of neglected and underutilised crop species and were not available in English. After that, the relevant articles were left and necessary data was extracted, the data were then subjected to bibliometric analysis. Bibliometric analysis is a quantitative method used to assess published articles and has become helpful to evaluate peer-reviewed studies in a specific field of research (Small, 1973; Lozano et al., 2019). The evolutionary trends were inferred from statistically assessing the occurrence and co-occurrence of key terms used to map trends in NUFMS using VOSviewer software. The titles and abstracts of articles in the final database (with 105 articles in Table 2) were used in the VOSviewer to investigate how concepts and topics have evolved over the years.

**TABLE 2:** Identified key experts and their role in the commercialisation process of medicinal plants.

Expertise	Role
Agronomist	To improve techniques for the cultivation of medicinal plants
Conservation campaigners	To persuade the public of the need to conserve medicinal plants
Ecologists	To understand the ecosystem in which medicinal plants grow
Ethnobotanists	To identify the use of plants as medicines in traditional science
Health policy-makers	To include conservation and utilisation of medicinal plants in their policy and planning
Horticulturists	To cultivate medicinal plants
Legal experts	To develop effective legal mechanisms that ensure that the collection of medicinal plants is at sustainable levels
Manufacturers	To develop processed products and by-products such as pills, lotions ointments
Nurseries	To produce and supply propagules and seedlings of medicinal plants
Pharmacognosists	To study the application of medicinal plants
Plant breeders	To breed improved strains of medicinal plants for cultivation
Plant genetic resources specialist	To assess and map the genetic variation in medicinal plants and maintain seed banks of medicinal plants
Plant pathologist	To protect the cultivated medicinal plants from pests and diseases without using dangerous chemicals
Seed biologists	To understand the germination and storage requirements of the seed of different medicinal plants
Taxonomist	To identify medicinal plants accurately
Traditional health practitioners	To provide information on the use and availability of medicinal pants

## 3 Results and Discussion

### 3.1 Conceptual Definitions of Key Terms

According to Blum (2016), “correct definitions and use of terms are not just a matter of formality but is essential for emerging research.” Consensus in the use of terms and definitions determine the success or failure of research, and the lack of it undermines attempts to reproduce the specific outputs, hence outcomes (Blum, 2016). Currently, there is an array of terms used in defining the medicinal properties of crops, each with a different meaning and context, and this has tended to work against NUFMS. In this section, we define and explain key terms that have been used to describe

#### 3.1.1 Medicinal Plants

A medicinal plant contains active compounds or therapeutic properties in one or more of its organs, which can be pharmacologically beneficial to the human body (Rasool, 2012; Namdeo, 2018). Medicinal plants, also called medicinal herbs, have been discovered and used in traditional medicine practices since prehistoric times. Medicinal plants are widely used in non-industrialised societies, mainly because they are cheaper than modern medicines. Currently, the science of medicinal plants has been recognised as “alternative medicine” where synergetic effects and preventative properties have been studied (Rasool, 2012).

#### 3.1.2 Functional Food Crops

Functional foods are defined as “a food category in which the products are either modified or fortified with substances that have a preventive or therapeutic effect beyond their original nutritional value” (Jonas and Beckmann, 1998). Functional foods can also be defined as “foods that are similar in appearance to a conventional food and possess physiological benefits or reduce the risk of chronic disease beyond basic nutritional functions” (Sawahla, 2014). Functional foods should be consumed at sufficient levels to positively impact the body (Vattem and Maitin, 2015).

#### 3.1.3 Neglected and Underutilised Crops

Neglected and underutilised crop species (NUS) refer to crop species that were primarily grown in their native communities but are currently losing their popularity (Padulosi et al., 2002). These crops have significant potential as food and industrial crops but are marginalised, if not entirely sidelined, by researchers, breeders, policymakers, producers and traders (Mabhaudhi et al., 2017). Consequently, they have poorly developed and understood value chains. They are non-commodity crops and belong to a large, biodiverse group of domesticated, semi-domesticated or wild species and, in most instances, are locally adapted (Padulosi et al., 2013). They are cultivated in traditional systems, mostly under subsistence level, while using informal seed systems (Dansi et al., 2012). Due to the need to increase agricultural productivity, NUS have been receiving attention because of their potential in promoting food and nutrition security in marginal areas.

#### 3.1.4 Dietary Supplement

Dietary supplements are another major nutraceutical class that includes concentrated food-derived nutrients (Phillips and Rimmer, 2013). Dietary supplements are not intended to replace food but are designed to provide added nutrients or perceived health benefits to daily food consumption.

#### 3.1.5 Pharmaceuticals

Pharmaceuticals are therapeutic and biologically active substances used to treat diseases (Taylor, 2015). They are structures that makeup drugs that can be complex or simple aromatic molecules (Taylor, 2015). Pharmaceuticals can be packaged as capsules, tablets, liquid or gel, and the quantity taken should be within a prescribed limit; otherwise, effectiveness will be diminished.

#### 3.1.6 Nutraceuticals

Nutraceuticals refers to the science dealing with the bioactive plant-based compounds that are isolated, packaged to be distributed in medicinal form and consumed to alter or maintain normal body functions (Das et al., 2012; Verma et al., 2017). The term also includes whole plants providing nutritional supplementation, such as honeybush tea, which provides antioxidants. The term “nutraceutical” was derived from “nutrition” and “pharmaceutical”. Nutraceuticals can be used as part of the diet to prevent or treat diseases. Some countries have not adopted the term “nutraceutical”; hence other terminologies are used to describe them in that regard. For example, in the United States of America, they are labelled as “dietary supplements” while in India, they are referred to as “foods for special dietary use”. In South Africa, the term “nutraceuticals” is not recognised in its drug-control law; they are referred to as “complementary medicine” and regulated by the South African Health Products Regulatory Authority (SAHPRA).

### 3.2 Characteristics of Searched Literature

#### 3.2.1 Diversity of Functional Medicinal NUCs

The NUFMS in this study were of nutritional or medicinal significance being used to treat specific ailments within communities. The study listed 226 plants belonging to 105 medicinal species sampled worldwide (Table 1) but found in South Africa. The medicinal plants pooled were of use in 29 countries. The regions represented by the sampled articles included West Africa (32%), North Africa (4%), East Africa (2%), Southern Africa (6%), Asia (47%), North America (3%), South America (1%), Europe (4%) and the Middle East (1%). The plants included legumes (16), pseudocereals (4), cereals (9), cucurbits (7), herbs (9), leafy vegetables (10), roots and tubers (10), shrubs (14) and trees (9). The plants presented a variety of growth behaviours, and the parts utilised also varied.

From the literature review results, South African research has focused on seven of the 105 neglected and underutilised functional medicinal crops. These include honeybush (*Cyclopia* Vent.), drumstick (*Moringa oleifera* L.), carob (*Ceratonia siliqua* L.), cancer bush (*Sutherlandia frutescens* L.), wild ginger (*Siphonochilus aethiopicus* (Schweinf.) B.L.Burtt), bitter gourd (*Momordica charantia* L.) and cactus pear (*Opuntia robusta* H.L.Wendl. ex Pfeiff*.*). Among these, the honey bush was the most frequently cited plant, appearing in five of the 105 articles of the sampled pool. Collectively, the plants had medicinal uses as antioxidants, anti-inflammatory and anti-cancer properties. Other ailments and conditions treated included arthritis, diabetes, helminthiasis, liver problems and fever. Nutritionally, the plants were utilised as appetite stimulants and a source of minerals. The plants include legumes (honeybush), shrubs (carob, cancer bush, cactus pear), root and tuber (wild ginger), tree (drumstick) and cucurbit (bitter gourd) (Table 1).

#### 3.2.2 Main Research Themes Coming out of the Literature

**FIGURE 2:**
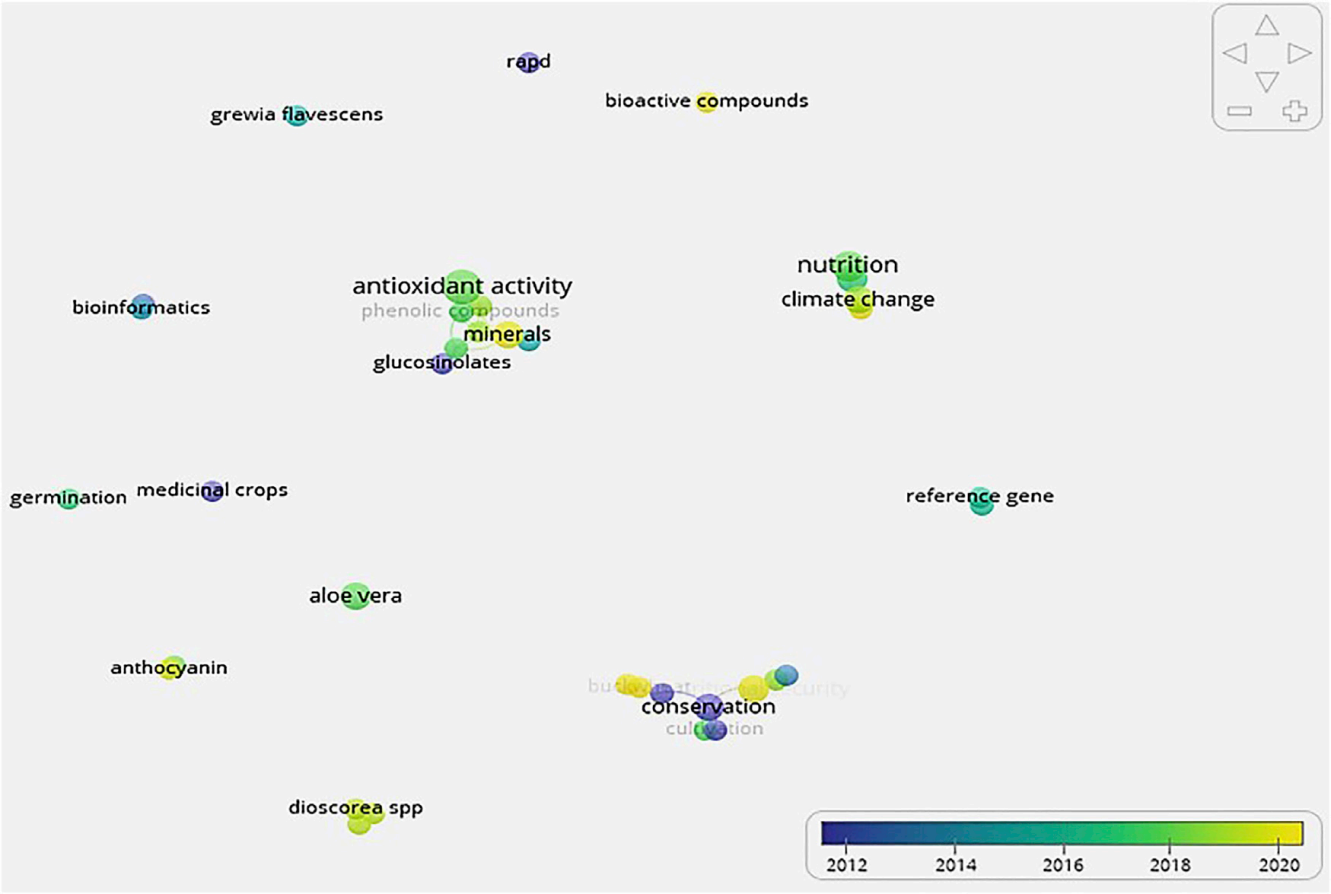
Direction and revolution of topical concepts on neglected and underutilised functional medicinal crops (NUFMS) derived using data from titles and abstracts in VosViewer.

**FIGURE 3:**
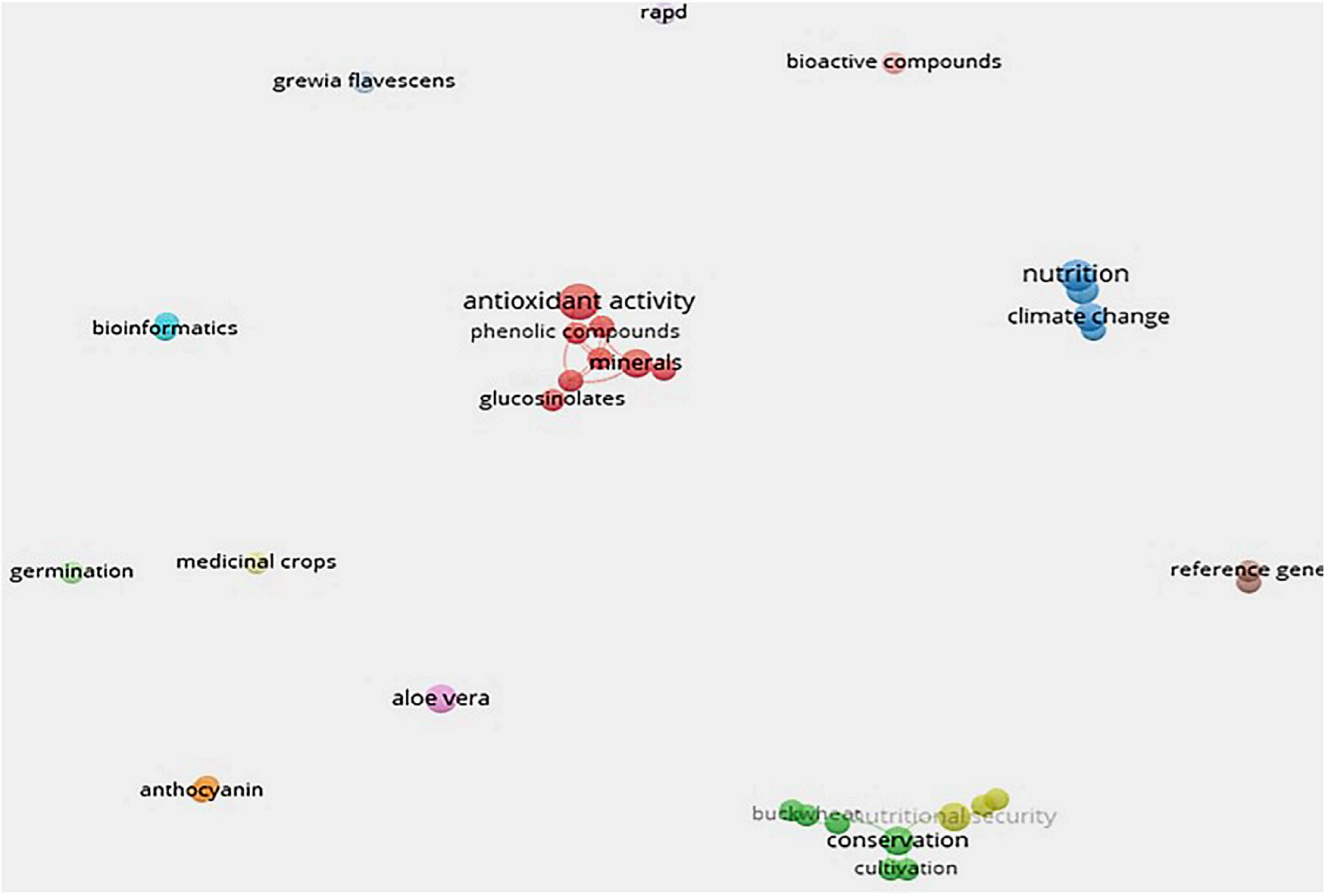
Topical concepts on neglected and underutilised functional medicinal crops (NUFMS) derived using data from titles and abstracts in VosViewer.

In evaluating the sourced literature on NUMFS, results (Figures 2, 3) showed that research into nutraceutical and pharmaceutical properties started trending globally in 2012, mostly in China and south-east Asia. During this period, the emphasis was placed on conservation. From 2014 to 2015, the period witnessed a shift from conservation to outlining the potential benefits of these crops (Figure 3). More recently, studies have focused on understanding the underlying mechanisms for the purported benefits. A closer look at the keywords showed three main thematic areas coming out of the source literature (Figures 2, 3).

The first thematic area, denoted by the red cluster, comprised terms that highlighted the biochemical properties of NUFMS (Supplementary Table S1). The thematic area represented the largest cluster; the keyword antioxidant activity had the highest citation (Figure 3). The compounds in this cluster associated with antioxidant properties of NUFMS included phenolic compounds, minerals, carotenoids, glucosinolates and proteins. Glucosinolates and proteins are located on the cluster’s periphery, indicating their role as indirect antioxidants. Glucosinolates stimulate natural antioxidant systems in the body while proteins work through inhibiting lipid oxidation. Purslane and amaranth were the only NUFMS frequently cited in the pool of research sampled for the network mapping.

The second thematic area denoted by the green cluster showed that research was still focusing on the sustainable use and production of NUFMS. The keyword “conservation” was mostly cited in articles that fell into this cluster, followed by tissue culture (Figure 3 and Supplementary Table S1). Tissue culture has been used to clone desirable NUFMS genomes, including buckwheat. Buckwheat is one of the most genetically diverse NUFMS, with 10,000 accessions currently stored in various gene banks globally (Chauhan et al., 2010). In addition, its popularity has increased, based on its diverse properties, which has enabled it to be used for nutritional security, medicinal purposes, industrial and as a cash crop (Suvorova, 2016). Similar research efforts are being made to document the NUFMS in different world regions; this is one of the crucial steps in their developmental journey. For instance, tissue culture technology has opened extensive research areas for micro-propagation, secondary metabolite production and biodiversity conservation for medicinal and endangered crop species (Tasheva and Kosturkova, 2012). Other biotechnological tools such as cryopreservation and genetic transformation are important to select, multiply and conserve the critical genotypes of medicinal plants. Cryopreservation is a long-term conservation method in liquid nitrogen and allows for the conservation of endangered medicinal plants (Wang et al., 2021). Genetic transformation may be a powerful tool for enhancing the productivity of novel secondary metabolites (Narayani and Srivastava, 2017). Genetic improvement of NUFMS is also currently underway (Jayaramachandran et al., 2020). Efforts are being made to genetically engineer sesame (*Sesamum indicum* L.) to improve its productivity through mutagenesis (Jayaramachandran et al., 2020). Sesame production has been affected by inherently low productivity; the genetically modified mutant has managed to produce larger seed weight and higher seed yield. DNA technology has also been used to ascertain, confirm and trace plant origins and phylogenetic associations. Barcoding using DNA has been used to ascertain the genealogy of some legumes such as lablab [*Lablab purpureus* (L.) Sweet], marama bean [*Tylosema esculentum* (Burch.) Schreiber] and cowpea [*Vigna unguiculata* (L.) Walp] (Popoola et al., 2019).

The blue cluster, which represents the third thematic area, is associated with the role of NUFMS in food and nutritional security. The keywords in this cluster included nutrition, food security, climate change and intercropping (Figure 3 and Supplementary Table S1). The research in this cluster indicates a response to climate change impacts on food production systems to ensure food and nutritional security. Intercropping, therefore, has been suggested as an intervention. According to Mabhaudhi et al. (2017), a key strategy to adapt to a changing climate is developing and promoting underutilised crop species (in our case NUFMS). Exploiting the large reservoir of underutilised crops would provide a more diversified agricultural system and food sources necessary to address food and nutrition security concerns under climate change (Mabhaudhi et al., 2019). Furthermore, there is vast potential for these crops to increase agricultural diversification and minimise environmental degradation; this has a direct impact on climate adaptation and mitigation (Beddington et al., 2011; Mabhaudhi et al., 2019).

The rest of the clustering was species dependant e.g. *Dioscorea* and bioactive compounds; safflower (*Carthamus tinctorius* L.) and gene sequencing. The studies falling into these clusters assessed (quantitatively and qualitatively) the properties of NUFMS using biotechniques (Figure 3 and Supplementary Table S1).

#### 3.2.3 Identified Crop Types and Their Distribution

##### 3.2.3.1 Legumes

The most cited leguminous plant included honeybush tea, velvet bean [*Mucuna pruriens* (L) DC.] and pigeon pea [*Cajanus cajan* (L.) Millsp*.*]. They are mostly utilised in India and Africa, growing as climbers or shrubs. Seeds are the most used parts in 60% of the legumes listed in this study. The legumes were used as dual-purpose plants to provide nutritional elements and medicinal use. Legumes such as tiger nut (*Cyperus esculentus* L.), bambara groundnut, grass pea (*Lathyrus sativus* L.), broad bean (*Vicia faba* L.) have been cited in the treatment of cardiovascular diseases, cancer, and diabetes in addition to being sources of nutritional compounds (Fasoyiro et al., 2006). Additional properties were reported in honeybush, where an increase in appetite, reduction in digestive problems, and relief for arthritis have been noticed in patients taking the tea. The elements provided for by legumes included crude fibre, crude fat, various amino acids, and mineral elements such as iron, calcium, magnesium, and phosphorus. Only ground bean [*Macrotyloma geocarpum* (Harms) Maréchal and Baudet] and pigeon pea were singularly used to provide nutrients (Table 1).

##### 3.2.3.2 Cucurbit

Cucurbits were popular in Asia (Korea, India, Iran, and China) and Africa (South Africa and Benin). Most plants in this category are creepers and have a dual function of providing nutrients and treating diseases except for *Cucumeropsis mannii*, bottle gourd [*Lagenaria siceraria* (Molina) Standl*.*], pumpkins (*Cucurbita pepo* L.) which provide nutritional compounds only. Their seeds and fruits provide nutrients such as vitamins, carbohydrates, fibre and minerals. The ailments treated by the listed cucurbits include bowel movements, helminthes, cancer and liver disease (Table 1).

##### 3.2.3.3 Leafy Vegetables

Leafy vegetables are mostly utilised in Africa (Benin, Ghana), Europe (Spain and Netherlands), Asia (India and Iran). Lamb’s quarters (*Chenopodium album* L.) and kale (*Brassica oleracea* L.) were the most frequently cited leafy vegetables. The leaves were primarily consumed, while in the case of common dandelion [*Taraxacum officinale* (L.) Weber ex F.H.Wigg*.*], jute mallow (*Corchorus olitorius* L.), black sesame (*Sesamum radiatum* Schumach. and Thonn*.*), lamb’s quarters’ seeds, roots and shoots were utilized (Table 1). They have been listed as sources of beta-carotene, phenolics, carbohydrates, fatty acids and minerals (Uusiku et al., 2010). The legumes were collectively used to treat diabetes, inflammation, hypertension, malaria, liver problems, helminths, cancer, haemorrhoids and indigestion. The spider flower (*Cleome gynandra* L.) is particularly used as an aphrodisiac.

##### 3.2.3.4 Cereals

This group is mostly associated with grasses, and their seeds are consumed primarily to provide carbohydrates. In addition, the seeds have been explored for containing lysine; minerals such as sodium, potassium, magnesium, manganese, phosphates, iron, zinc; vitamin A and B; phenolics and flavonoids (Table 1). The listed cereals were rarely used for medicinal purposes except for finger millet [*Eleusine coracana* (L.) Gaertn.], which treated some microbial infections and diabetes. In maize (*Zea mays* L.) and finger millet, shoots and leaves are consumed and their seeds. Cereals were reported in Africa (Benin and Kenya), Asia (India, Philippines, Iran, Vietnam) and America (Canada and United States). Maize and finger millet were the most frequently cited plants in the group.

##### 3.2.3.5 Pseudocereals

Six articles mentioned the use of pseudocereals as NUFMS in India (*n* = 2), China (*n* = 1) and unspecified geographical locations (*n* = 3). Buckwheat (*Fagopyrum esculentum* Moench.) and amaranth were used medicinally to treat eczema, while *Amaranthus spinosus* (L.) was a diuretic. Nutritionally, pseudocereals were sources of flavonoids, steroids, lipids, carbohydrates, crude fibre, amino acids, minerals, protein, ash, beta-carotene and phenolics. Parts utilised included seeds, leaves, flowers and shoots.

##### 3.2.3.6 Roots and Tubers

The geographical distribution of roots and tubers cited in articles selected for the study was confined to Asia (India and Philippines) and Africa (Ethiopia, Benin, South Africa, Tanzania). The most popular plant in the group was taro [*Colocasia esculenta* (L.) Schott] (*n* = 5; Table 1). Tubers were mostly consumed while the stem, leaves, shoots and corm were used in crops such as taro (Tattiyakul et al., 2006). Medicinal functions included use in the treatment of diarrhoea (*Dioscorea bulbifera* L.), ulcers [*Dioscorea esculenta* (Lour.) Burkill], kidney stones and appendicitis (taro). The yam [*Dioscorea dumetorum* (Kunth) Pax] has been used to treat or relieve many ailments associated with cancer, heart disease, diabetes, dysentery, inflammation, gonorrhoea, haemorrhages, and hypertension helminths. Nutritional contributions of roots and tubers included carbohydrates, crude fat, protein, crude fibre, minerals and phenolics (Table 1).

##### 3.2.3.7 Shrubs

Another popular class of NUFMS comprised plants classified as shrubs (*n* = 25). The plants were identified in Africa (South Africa, Ghana, Sri Lanka, Niger) and Asia (India, China, Philippines). Dual-purpose species used for both medicinal and nutritive purposes included donkey berry (*Grewia flavescens* Juss), Ethiopian eggplant (*Solanum aethiopicum* L.) and miracle fruit [*Synsepalum dulcificum* (Schumach. and Thonn.) Daniell] (Hirakawa et al., 2014 and Table 1). Shrubs used for the sole purpose of treating ailments included cannabis (*Cannabis sativa* L.) for inflammation; cancer bush for cancer and diabetes; pea eggplant (*Solanum torvum* Schltdl.) for curbing bacterial diseases, and cassava (*Manihot esculenta* Crantz.) to clean wounds. Shrubs used to provide nutrition only included hibiscus or roselle (*Hibiscus sabdariffa* L.), which is a source of manganese, copper, molybdenum and ascorbic acid; African eggplant (*Solanum macrocarpon* L.) for provision of fats, carbohydrates, proteins, crude fibre, minerals and vitamin c; bitter eggplant (*Solatium insanum* L.) for provision of carbohydrates, vitamin A, vitamin C and phenolics (Table 1).

##### 3.2.3.8 Trees

Tree species considered were from many regions, particularly West Africa (*n* = 7), Southern Africa (*n* = 2), Asia (*n* = 4) and Southern Europe (*n* = 1) (Table 1). The group is home to drumstick, cited most frequently (*n* = 7) among all the plant species considered in this study. A wide variety of plant parts were used and of note was the baobab (*Adansonia digitate* L.), which had eight parts being utilised for medicinal and nutritional purposes. These parts included fruits, seeds, roots, bark, stem, leaves, flowers and shoots (Table 1). Trees were used to treat diseases and conditions such as cancer, blood pressure, ulcers, inflammation, diabetes, diarrhoea, malaria and fever. Nutritional contribution by this group of plants included the provision of fats, carbohydrates, proteins, crude fibre, minerals and vitamins.

##### 3.2.3.9 Botanical Drugs/Herbs

Plants classified as herbs were mentioned in articles mainly from Asia. Fruits, seeds, leaves, flowers and roots were used to utilise their medicinal and nutritional properties. Most herbs listed worked exclusively as either medicinal or nutritional. Medicinal herbs included lemongrass [*Cymbopogon flexuosus* (Nees ex Steud.) Will. Watson] and bush tea (*Athrixia phylicoides* DC.) for diabetes, improving bowel movements, enhancing sexual potency and as a diuretic substance and water hyssop [*Bacopa monnieri* (L.) Wettst.] to enhance memory. Herbs used for nutritional purposes were fennel flower (*Nigella sativa* L.) and ladies’ fingers or okra [*Abelmoschus esculentus* (L.) Moench]. Dual-purpose herbs included safflower (*Carthamus tinctorius* L.) used as a source of flavonoids and for treatment of diarrhoea, cardiovascular diseases, helminths and diarrhoea and plantain (*Plantago major* L.) used as a source of phenolics and for treatment of cancer, viral diseases, diabetes, hypertension, inflammatory conditions, ulcers and cleaning wounds (Table 1).

### 3.3 Chemical Constituents of Functional Medicinal Crops

Plants have been used for thousands of years to flavour and conserve food, treat health disorders, and prevent diseases. Active compounds produced during secondary vegetal metabolism are usually responsible for the biological properties of some plant species used for various nutraceutical and pharmaceutical purposes. These active compounds also enhance their survival. These constituents have been identified as alkaloids (Varsha et al., 2013), glycosides (Firn, 2010), flavonoids (Varsha et al., 2013), phenolics (Puupponen-Pimiä et al., 2001), saponins (Vashist and Sharma, 2013), tannins (Varsha et al., 2013), essential oils (Canales-Martinez et al., 2008), and steroids (Madziga et al., 2010). In this section, we provide an overview of these constituents

*Alkaloids* contain highly active nitrogenous molecules and have anti-cancer and immune-stimulant properties (Shakkarpude et al., 2020). Examples of alkaloids found in plants include caffeine, theobromine and theophylline, classified as purine alkaloids and act as stimulants that increase heart function, vasodilation, and metabolism (Anaya et al., 2006). Alkaloids are being used to synthesise drugs, including vincristine, a common drug for leukaemia, made from Madagascar periwinkle (*Catharanthus roseus* (L.) G. Don) (Barrales-Cureño et al., 2019; Shakkarpude et al., 2020).

*Bitters*: The plants in this group have a characteristic bitter taste that stimulates the digestive system, including the salivary glands (Shakkarpude et al., 2020). As a result, bitters are considered effective as appetite-stimulants and ensure a well-functioning digestive system (Sahu et al., 2013)—examples of bitters are aloes, wormwood and hops.

*Cardiac Glycosides*: The group comprises steroids that work directly on the cardiac muscles. They have been used traditionally as poison and placed on arrows’ tips for hunting (Morsy, 2017). With advances in medical technology, drugs have been synthesised with the correct levels to treat heart diseases. In addition to cardiac muscle stimulating properties, cardiac glycosides are diuretics that assist in draining fluids out of the body (Shakkarpude et al., 2020). Examples of cardiac glycosides include digitoxin, digoxin and convallotaxin. Cardiac glycosides are found in the families Apocynaceae and Asclepiadaceae (Shakkarpude et al., 2020).

*Cyanogenic Glycosides*: Some plants, such as the wild cherry and the elderberry, produce hydrogen cyanide as a defence mechanism that relaxes the muscle, and in high amounts, it can be highly poisonous to humans (Yulvianti and Zidorn, 2021). Some examples of cyanogenic glycoside include amygdalin, which can be isolated from bitter almonds [*Prunus dulcis* (Mill.)]. In wild cherry and elderberry, the compounds are used to pacify coughs (Shakkarpude et al., 2020).

*Flavonoids*: Flavonoids are broadly found in nature and comprise secondary polyphenol metabolites with a wide range of medicinal properties (Wang et al., 2018). More than 9,000 types of flavonoids have been isolated from plants so far, and these are known to have anti-viral, anti-inflammatory, cardioprotective, anti-cancer, anti-ageing properties (Wang et al., 2018; Shakkarpude et al., 2020). Flavonoids can be found in various plants, including vegetables as quercetin and kaempferol (Ngameni et al., 2013). Chalcones, flavones, and isoflavones can be found in the *Dorstenia angusticornis* (L.) and cashew plant (*Anacardium occidentale* L.). Lemons also contain flavonoids and assist in strengthening the capillaries. They are responsible for colouring some plants characterised by red, blue, and purple pigments or yellow and white pigmentation on fruits and flowers (Ngameni et al., 2013; Shakkarpude et al., 2020).

*Minerals*: Minerals are acquired by plants from the soil hence are found in many plants. Minerals are responsible for ensuring general health, including disease prevention. Intake of sodium, potassium, magnesium, calcium, manganese, copper, zinc and iodine in the diet reduces the risk of cardiovascular disease (Sanchez-Castillo et al., 1998). Trace elements are significant in ensuring human metabolic processes as components of proteins such as haemoprotein and haemoglobin (Imelouane et al., 2011). Calcium is responsible for healthy teeth and bones, while sodium and potassium act as regulators. The horsetail plant [*Equisetum arvense* (L.)] is known to assist in repairing connective tissue.

*Phenols*: Phenolic compounds have chemopreventative properties, which enable them to be anti-carcinogenic (Huang et al., 2009). Examples of phenolic compounds found in herbal medicinal plants include phenolic acids, flavonoids, tannins, stilbenes, curcuminoids, coumarins, lignans and quinones. Apart from anti-cancer properties, phenolic compounds have anti-microbial properties that prevent infection (Shakkarpude et al., 2020). More than 8,000 phenolic compounds have been isolated from herbal medicinal plants, including wintergreen [*Gaultheria procumbens* (L.)], mint [*Mentha spicata* (L.)] and willow [*Salix alba* (L.)] (Tungmunnithum et al., 2018).

*Polysaccharides*: Polyssachaccharides are macromolecules that exist as structural molecules in plants. They exist in plants as monosaccharides in the form of starch and cellulose. Cellulose and hemicellulose are insoluble in water and bulky, and hence they assist in bowel movements. Other bioactivities of polysaccharides in herbal medicinal plants include anti-tumour activity, antioxidant activity, anti-coagulant activity, anti-diabetic activity, radioprotection effect, anti-viral activity, hypolipidemic immunomodulatory (Wang et al., 2008; Xie et al., 2016).

*Proanthocyanins* are compounds closely related to tannins and flavonoids, which give plants their colouring. The compounds have anti-inflammatory, antioxidant and anti-cancer properties hence are important in heart, eyes and feet health (Shakkarpude et al., 2020). Plants that contain large quantities of proanthocyanins include blackberries ad hawthorn berries. The bioavailability of proanthocyanidins is primarily influenced by polymerization, which is rendered insignificant in the gastric tract (Golovinskaia and Wang 2021).

*Saponins*: Saponins are secondary metabolites, surface-active glycosides with a characteristic foaming ability (Shakkarpude et al., 2020). Steroidal and triterpenoid are the two types of naturally occurring saponins. Their benefits include lowering cholesterol levels, treating diarrhoea and having anti-microbial properties (Desai et al., 2009). Plants containing high levels of saponins include agave, wild yam, and several lily family members.

*Tannins*: Tannins are polyphenols of a high molecular weight converted to quinones when oxidised (Pereira et al., 2015). Their bitter taste is part of the protective mechanisms in plants to protect them from herbivores. They are, however, utilised in processing leather during the tanning stage. They have a binding effect on proteins, thereby creating a protective layer that prevents microbes’ action (Pereira et al., 2015). They are highly concentrated in the bark, sap, fruits and leaves of plants such as oak bark [*Quercus* (L.)] and black catechu [*Senegalia catechu* (L.f.) P.J.H.Hurter and Mabb].

*Vitamins*: Vitamins are compounds with many uses and are required in small amounts to maintain a constant body environment. Antioxidant vitamins, particularly Vitamin A, C and E, scavenge for free radicals in the body. Fruits and vegetables are sources of vitamins, while plants such as rose hips [*Rosa canina* (L.)] and sea buckthorn [*Hippophae rhamnoides* (L.)] are rich in vitamins B, C and E. Ascorbate or vitamin is also a cofactor for ascorbate peroxidase (Smith et al., 2007) and phylloquinone or vitamin K is involved in the electron transport chain.

*Volatile oils*: Volatile oils are highly complex oils extracted from plants and are used to produce essential oils. They contain more than 100 compounds (Shakkarpude et al., 2020). Essential oils have anti-microbial, anti-carcinogenic, anti-diabetic and antioxidant properties (Reddy, 2019). *Apiaceae, Lamiaceae, Myrtaceae, Poaceae*, and *Rutaceae* families are significant sources of essential oils.

*Antioxidants*: are molecules that inhibit the production of free radicals in the body through oxidation. Compounds that act as antioxidants include flavonoids, phenolics, sterols, alkaloids, carotenoids and glucosinolates (Saranya et al., 2017). Medicinal plants such as turmeric (*Curcuma longa* L.), cinnamon (*Cinnamomum verum* L.), onion [*Allium sativum* (L.)], ginger [*Zingiber officinale* (Roscoe.)], safflon (*Crocus sativus* (L.)]*,* hopbush [*Dodonaea viscosa* Jacq. subsp. *angustifolia* (L.f.) J.G. West], Barleria noctiflora [*Barleria noctiflora* (L.)]*,* cashew tree [*Anacardium occidentale* (L.)], Indian Thornapple [*Datura fastuosa* (L.)] and fever nut [*Caesalpinia bonducella* (L.) Fleming] are among many other plants that have antioxidant properties.

### 3.4 Pharmacological Properties

**FIGURE 4:**
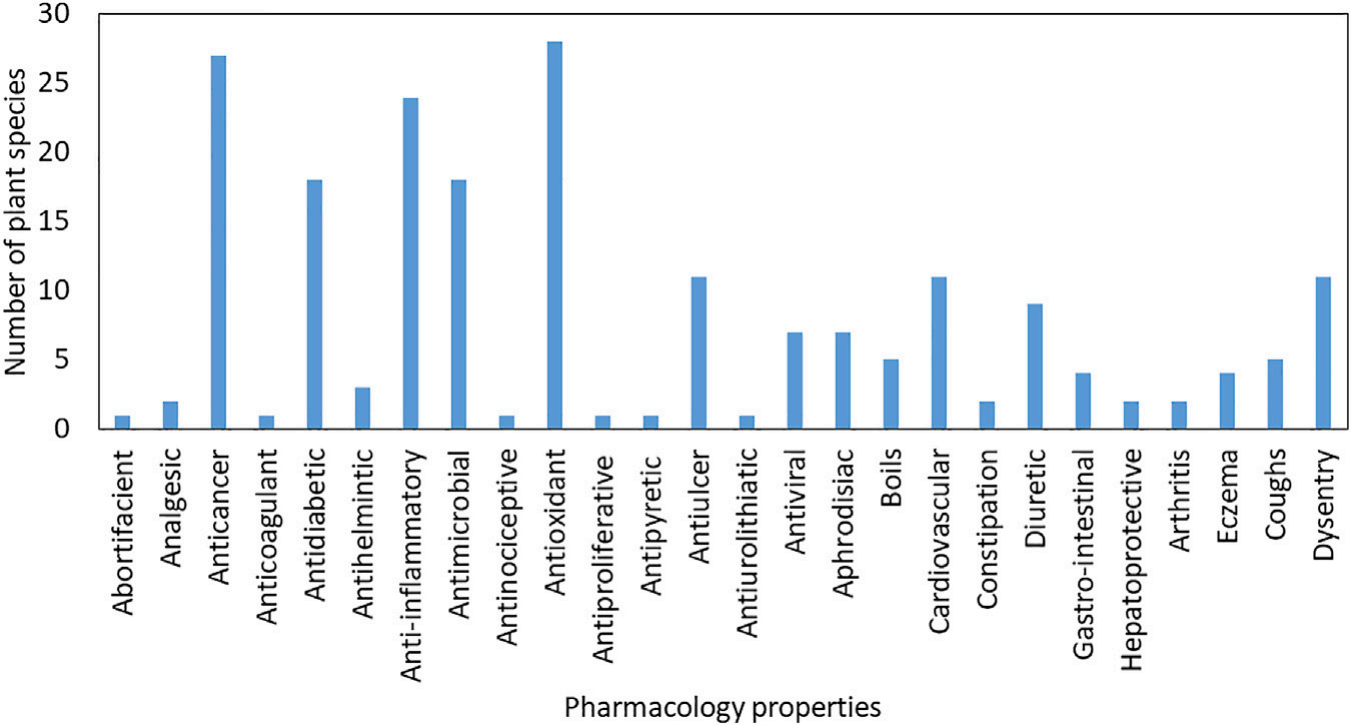
Total frequency of articles citing pharmacological properties possessed by neglected and underutilised functional medical crops found in South Africa.

Studies to determine herbal medicinal plants’ chemical profile and composition reveal the complexity and variety of compounds contributing to plants’ various uses in treating numerous aliments, including life-threatening diseases such as HIV/AIDS, cancer, cardiovascular, and diabetes. Sexually transmitted infections (STIs) are among the most common reasons people use herbal medicines and visit traditional healers in South Africa. NUFMS plays an important role in providing nutrition and treating chronic diseases, especially in many indigenous communities and developing regions. Neglected and underutilised functional medicinal crop species exhibit various pharmaceutical properties that include, but are not limited to, anti-inflammatory, anti-spasmodic, antioxidative, anti-bacterial, anti-fungal, anti-cancer, anti-allergic, hypoglycemic, analgesic, immunomodulatory, anti-stress, anti-ulcerogenic, anti-hypertensive, hepatoprotective, chemopreventive, radioprotective, anti-tumour, and anti-pyretic (Figure 4). The majority of the plant species identified (91.3%) had multiple pharmacological uses, with 45 species (42.4%) used in the treatment of five or more ailments, 18 species (16.5%) treating between five and three ailments, and 15 species (11.5%) treating three or fewer ailments (Table 1). Gastro-intestinal disorders, STIs, cold, cough and sore throat and gynaecological problems were treated with using most of the identified herbal medicinal plant species (Table 1). Gastro-intestinal disorders, particularly cholera, diarrhoea, and dysentery, are a major concern in South Africa and the whole region (Ribeiro et al., 2010) due to poor access to clean water, sanitation and hygiene. Sexually transmitted infections are a major public health concern in developing countries, with their transmission rate regarded as one of the highest in the world (Van Vuuren and Naidoo, 2010).

## 4 Production Strategy for Neglected and Underutilised Functional Medicinal Crops

The NUFMS industry is considered an “uncharted” economy dominated by local production and market systems, which are less organised. In response to high population growth rates, rapid urbanisation and the important cultural value placed on traditional medicines and health remedies, the demand for these plants is expected to grow (Mander et al., 2007). Currently, the demand for NUFMS plants out-strips supplies. This is evidenced by the high level of over-harvesting and extinction from the wild and resultant high prices in the market. Most crop species harvested from the wild have been considered endangered herbal medicinal plant species. On the other hand, low yielding potential has meant low productivity due to low genetic and breeding focus and poor agronomic practices. The depletion of wild stocks and low productivity have been attributed to the unsustainable access and availability of NUFMS. Concurrently, it has now been recognised that their conservation and production should be coupled with poverty alleviation initiatives for rural communities. There is a growing need to increase research and development on NUMFS since they are 1) an important plant genetic resource, 2) a resource for the synthesis of natural medicines and nutrients, and 3) support the health and nutrition of poor South Africans. The motivation to conserve and cultivate these plants should involve a socio-economic and technical approach in which attention is given to the synergistic interlinkages between economic, technical, cultural and institutional dimensions of domestication. Key activities will thus be used to formulate a strategic business plan. These will include conservation and propagation, research and development, marketing and distribution and policy.

Since the development of NUFMS plants in South Africa is at its infancy, understanding their current status using a value chain approach will be considered in developing a guideline for the commercialisation of NUFMS plants.

### 4.1 Understanding the Status of Functional Medicinal Crops

**FIGURE 5:**
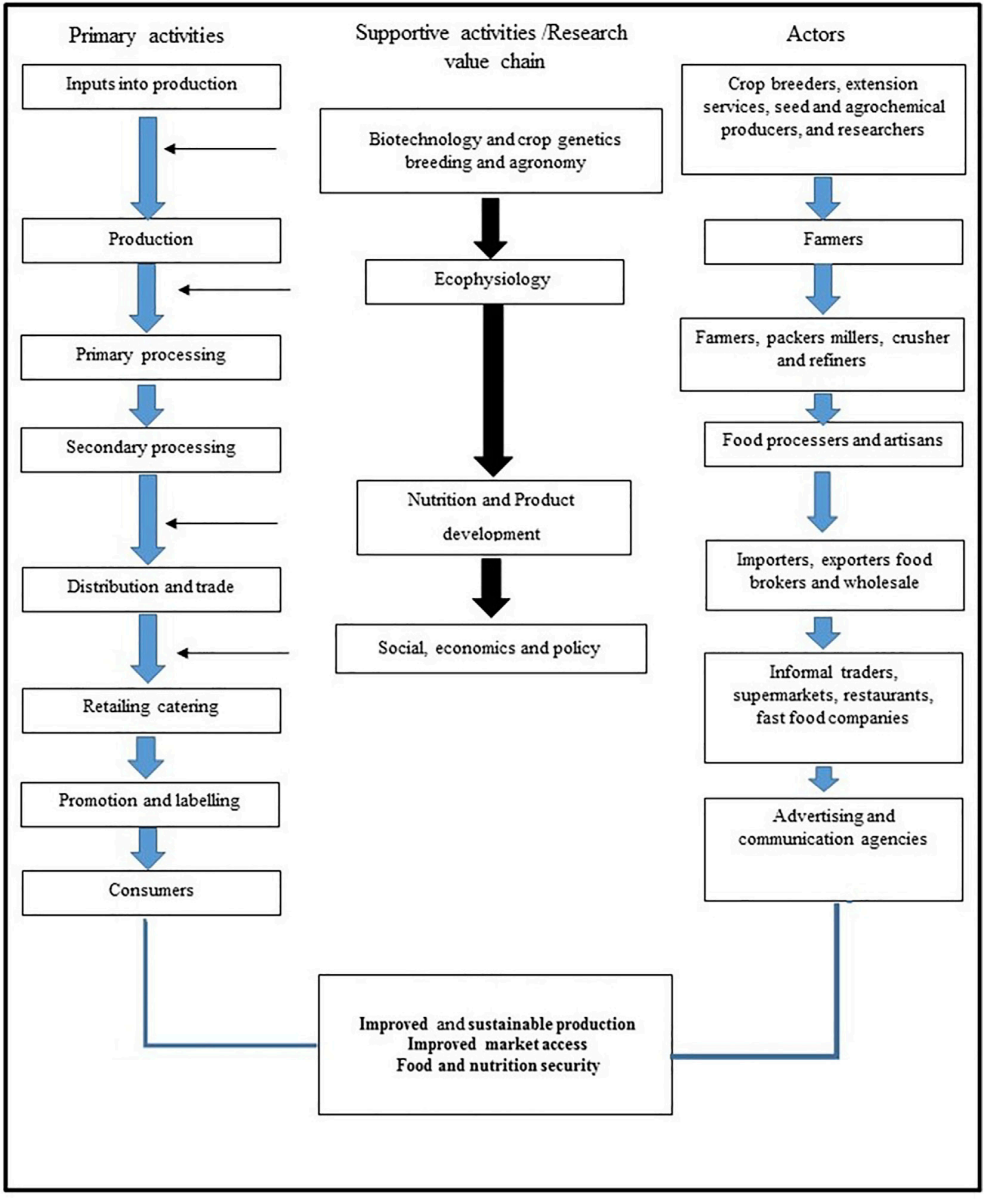
Value chain of neglected and underutilised crops indicating primary and support activities and actors involved during the primary activities (Source: Mabhaudhi et al., 2017).

There is a need to improve information, the supply, marketing and production of functional medicinal plant material (Akinola et al., 2020). This is because wild plants are under extreme pressure due to increased demand from local and export markets, and the productivity of many of these crop species remains far too low for meaningful economic returns (Sivakumar et al., 2004; Tasheva and Kosturkova, 2012; Mabhaudhi et al., 2017; Akinola et al., 2020). In addition, climate variability and change have resulted in reduced availability and potency of these crop species owing to unfavourable growing conditions (Mabhaudhi et al., 2017). Therefore, the status of functional medicinal crops in SA was reviewed to identify existing gaps, opportunities, and challenges for developing future research capacity. It is hoped that a framework to transform these crops into a commercially viable enterprise can be developed through understanding the status. Also, within the context of SA, we can identify priority herbal medicinal crops. This can be achieved through the research value chain, i.e. breeding/crop improvement—production—agro-processing—marketing (Figure 5).

A value chain refers to the “full range of activities which are required to bring a product or service from its commencement, through the different phases of product delivery to final consumers, and final disposal after use” (Padulosi et al., 2002). Such an approach was considered ideal for the development of herbal medicinal plants. It would allow for a holistic strategy that gave equal focus on all aspects required to commercialise herbal medicinal plants (Gómez and Ricketts, 2013; Manyise and Dentoni, 2021). In the context of rural agricultural development, value-chain approaches have since become a popular strategy to encourage greater participation in national and international markets (Sharma and Chen, 2020). Such a strategy has also been proposed to promote lesser products such as NUFMS (Padulosi et al., 2002); however, little has been done to develop sustainable value chains. Value chains of NUFMS remain poorly developed, with few activities and actors (Tran et al., 2013). There is a need to identify actors and their key roles to successfully transform NUFMS into commodity crops. Figure 5 proposes a simple food value chain. It outlines the “who”, “when”, and “how” considerations to guide scaling NUFMS value chains. Support activities play a pivotal role in identifying and enhancing the “value” that must be added, thus incentivising a broader spectrum of actors (Tran et al., 2013). This set of activities has been coined the “research value chain” (Figure 5).

**FIGURE 6:**
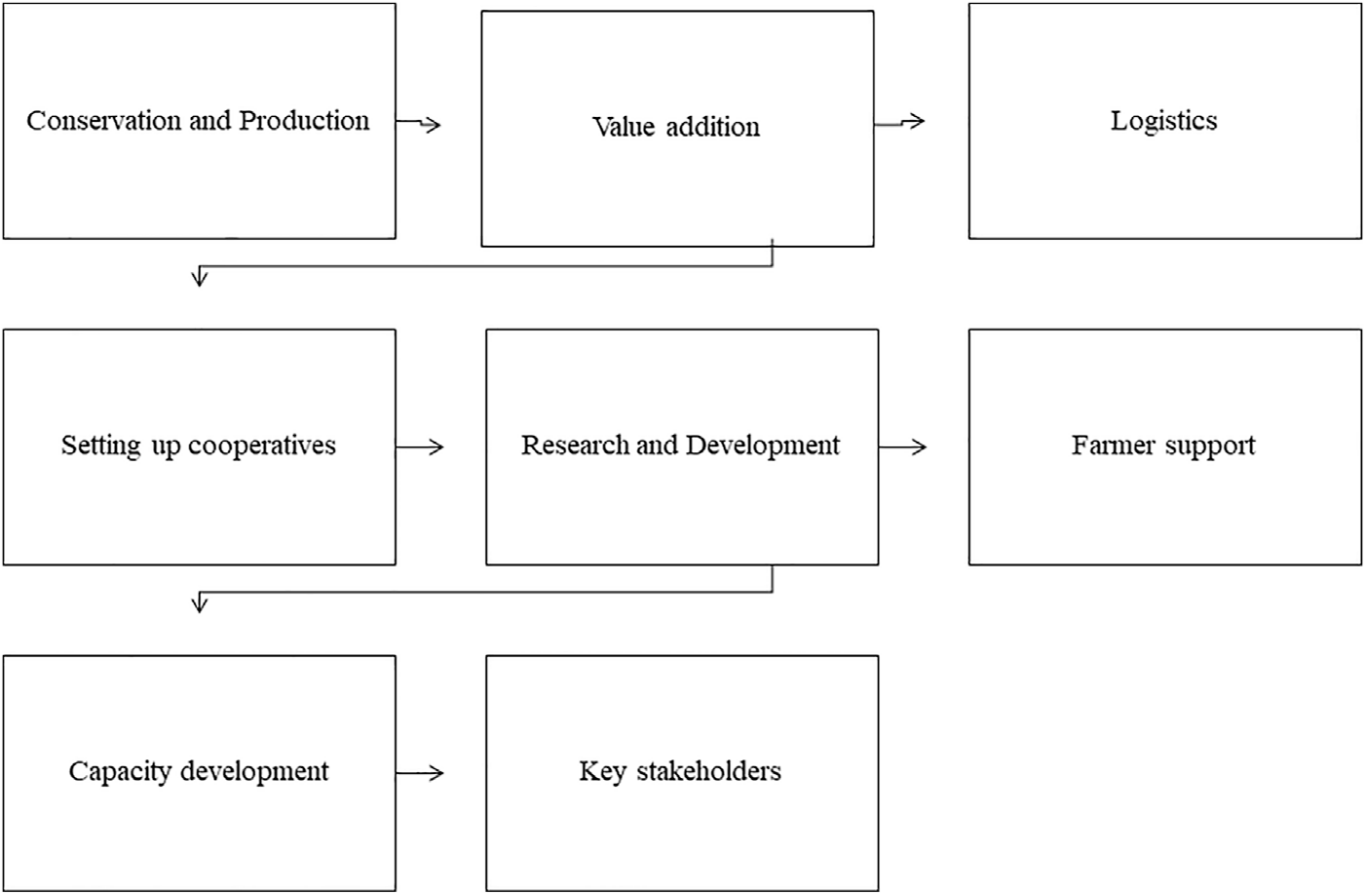
Production strategy for neglected and underutilised functional medicinal crops.

The value chain approach is also accepted for developing and promoting emerging technologies across different sectors (Sharma and Chen, 2020). The lack of an explicit focus on the supportive role of research, development and innovation (RDI) may partly explain the slow progress in developing NUFMS value chains. Research, development and innovation are needed to generate evidence, and integrate indigenous knowledge; much information on NUFMS currently resides in indigenous knowledge systems. A clear and targeted RDI roadmap could unlock the potential of NUFMS. As an entry point, there is a need to consider key activities for sustainable production and use of these crop species (Figure 6).

### 4.2 Conservation and Production

The initial point should be at the start-up point of the value chain, i.e., for successful and sustainable commercialisation of NUFMS to occur, the supply side has to be developed. This entails shifting from current practices of harvesting wild stocks, which are already showing signs of depletion and focussing on conservation, domestication and sustainable cultivation of herbal medicinal plants. In this regard, the following is therefore proposed:

*Short-term*: in the short term, there is a need to introduce conservation strategies for herbal medicinal plants, especially those flagged as vulnerable or near endangered. These strategies will ensure consistent plant genetic resources to expand the medicinal plant’s industry. Therefore, collectors of NUFMS should be educated on sustainable harvesting practices (Box 1). As part of the strategy, *in situ* conservation, which entails on-site conservation through improved forms of controlled use of naturally growing plants, should be explored.

*Medium-term: ex-situ* conservation, i.e., off-site conservation, where part of the population is removed from a threatened habitat and placed in a new location, should be explored. This will allow for the regeneration of declining wild stocks and provide new material to meet demand while relieving wild stocks. This stage also involves the setting up and/or establishing dedicated medicinal plants nurseries in areas where targeted farmers are located.

*Long-term:* as a long-term strategy to conservation and meet demand, *in-domo* conservation should be explored. This would involve conservation by developing cultivation practices to conserve herbal medicinal plant species. *In-domo* conservation will form the basis for promoting the cultivation of herbal medicinal plants within smallholder farming communities across the country. This stage will require support from RDI, policymakers, civil society and community engagement to co-develop and co-implement sustainable agricultural intensification practices.

### 4.3 Value Addition

Value addition is often the mid-point of the value chain and includes activities from the farm gate up to distribution. It includes activities such as post-harvest handling and storage as well as agro-processing. The objective of this stage is to add to the value of the raw material and improve its utilisation. For developing countries and rural economic development, this is a key point of the value chain, which remains under-developed and may explain why farmers continue to earn less from their production. It also provides the most opportunities for setting up new agro-processing industries in rural areas, product development and supporting inclusive rural economic development. The following should be noted:

• Within the context of medicinal plants, agro-processing refers to the set of techno-economic activities carried out for conservation, production and handling of plants to make them usable as herbal medicines, food supplements and/or industrial raw material;
• Agro-processing of these plants includes all operations from the stages of cultivation, harvesting and post-harvest handling until the material reaches the end-users in the desired quality;
• Developing agro-processing capacity for NUFMS will certainly have a significant impact on the sustainable supply and marketing of a wide range of plant products;
• There is a lack of local information describing the pharmacology, post-harvest handling and storage information for the range of plants currently utilised in South Africa;
• As a starting point, this review recommends the adoption of the World Health Organisation (Organización Mundial de la Salud, and World Health Organization, 2003) guidelines on Good Agricultural and Collection Practices (GACP) and post-harvest processing for NUFMS;
• Due to the lack of robust and empirical information describing the pharmacology and post-harvest handling and storage of NUFMS, there is a need for RDI investments in this area; and
• While some knowledge already exists in indigenous knowledge (IK) mostly held by traditional healers and herbalists, this IK remains poorly documented. Research and development should initially seek to document this IK and then integrate it with scientific knowledge. The integration of IK and scientific knowledge will open new and inclusive pathways for allowing traditional healers and herbalists to participate in the knowledge economy.

An Agri-hub should be set up to address the agro-processing aspect of the value chain. However, it is critical to note that the success of the Agri-hub depends largely on RDI investments into the entry point of the value chain (i.e., conservation, propagation and propagation) as well as components of the mid-point value chain that have been discussed above.

### 4.4 Logistics

Logistics management is a cross-cutting aspect of value chains involved in organising, controlling and implementing operations along the value chain. It also manages the forward and backward interactions of the value chain. In this regard, logistics planning plays a critical role in the viability and sustainability of any value chain. Since value chains for herbal medicinal plants are still under-developed, the following logistical considerations should form part of the strategy:

*Pre-production*: there is a need to establish nurseries for priority NUFMS plants within suitable sites. These will be used as a source of plant material for supplying identified farmers and encouraging cultivation;

*Production*: there is a need to invest in infrastructure such as tunnels/greenhouses to support all-year round production of NUFMS plants and thus guarantee the stability of supply;

*Technology*: there is a need to invest in sustainable and efficient irrigation technologies as most environments in which NUFMS grow are water-scarce areas. To mitigate increasing demand for water and energy, complementary technologies such as solar pumps, rainwater harvesting and conservation and use of grey water, could be considered. This will contribute to the overall resilience of production;

*Input support*: in the initial phase, there is a need to support identified farmers with inputs, i.e., planting material, fertilisers, herbicides and pesticides. This will ensure yield potential is realised and that quality material is also produced. Input support should also be complemented with farmer training so that over time farmers are capable procuring own inputs and managing the enterprise; and

*Post-harvest*: integrated pre-and post-harvest management practices should be developed for identified priority NUFMS. Like nurseries, at least adequate packhouses should be established. Low-cost water and energy efffcient technologies such as air and solar drying and biogas digesters should be prioritised as these can easily be set up within rural communities. This will ensure that quality established during field production is maintained throughout the value chain.

### 4.5 Setting up Cooperatives

The logistical considerations also give rise to opportunities or setting up cooperatives to address various aspects of logistics as follows:

*Primary co-ops*: these will be related to managing logistics linked to the start-up/entry point of the value chain. These activities include the production of herbal medicinal plants. As such, smaller cooperatives that offer geographic and economies of scale advantages, in terms of land area, could be explored as a starting point.

*Secondary co-ops*: could target common/shared interest areas within the appropriate location. Given that part of the strategy recommends setting up nurseries in target areas, this could be a point of formation combining the various primary co-ops in each of the areas to form a secondary co-op for managing the nurseries, and

*Tertiary co-ops:* this is high level and specialised co-op which will possibly manage the procurement of inputs, aggregation and distribution logistics (refrigerated trucks and packhouses).

### 4.6 Research, Development and Innovation for Herbal Medicinal Plants

Research, development and innovation on herbal medicinal plants is still limited due to the historical neglect of RDI investments and prioritisation that favoured a few major crops. Successful commercialisation of medicinal plants requires that RDI on NUFMS be focused on unlocking and supporting points in the value chain. In this regard, RDI on the chemical properties and value of plants and their extracts and the development of commercial, pharmaceutical, and nutritional products that may arise from the chemical analysis is required. In addition, indigenous knowledge systems linked to plants and their uses and the ecological and economic considerations relating to production should be documents and integrated with scientific knowledge. Activities that can lead to achieving these goals include:

• Continuous research into the agronomic considerations and conservation of priority medicinal plants.
• Research on traditional knowledge and medicine systems.
• Research op agro-technique, biodiversity, biotechnology and genetic improvement medicinal plants. This includes biosynthesis and metabolic assays, tissue culture and propagation, phytochemical research; plant-derived agents for cancer, immune-related diseases and hepatotoxicity
• Quality control assessment and research on active ingredients and substances of NUFMS.

### 4.7 Agribusiness Accelerators

There is a strong case to provide technical and agribusiness support for the farmers along with the various points of the NUFMS value chain through establishing dedicated Agribusiness Accelerators (AAs) consisting of transdisciplinary stakeholders (researchers/academics, government, private sector, civil society, communities/farmers, and traditional healers/herbalists). The role of the AAs will be as follows:

• To co-develop and co-implement an RDI roadmap for supporting the NUFMS value chain;
• To provide monitoring and evaluation support and capacity for interventions and investments made in targeted areas;
• To provide technical support geared at propagation, production, post-harvest handling and storage;
• To support the establishment of production enterprises by providing information on best management practices related to sustainable conservation and production of NUFMS, and
• To oversee capacity development of farmers by offering regular training and field visits and farmer field days. The AAs will initiate knowledge development concerning the production of NUFMS.

The role of the AA is both strategic and critical to the unpacking of any strategy, and guiding the sustainable commercialisation of NUFMS. As such, the formation of the AAs should be prioritised as it is catalytic to the overall success of the NUFMS RDI Roadmap.

### 4.8 Capacity Development

The lack of knowledge transfer between researchers and farmers, extension services policymakers and lobbyists is widely recognised. This is also indicative of a lack of transdisciplinarity and knowledge co-creation and co-implementation. This knowledge bottleneck may affect the successful commercialisation of herbal medicinal plants. Researchers need to translate, package, and transmit their findings for information regarding medicinal plants to reach intended beneficiaries. Research—training—deployment constitutes a 3-dimensional capacity development approach that can increase knowledge skills and experiences in the herbal medicinal plant industry. Capacity development is how individuals and organisations obtain, strengthen and maintain the capabilities to set and achieve their development objectives over time. In this regard, capacity is about the growth of individuals and/or institutes in herbal medicinal plants’ knowledge, skills, and experience. For this to happen, the following is proposed

*Research*: Database containing medicinal plants should be created and accessible to herbal medicinal plant stakeholders. The development of such a database should involve all stakeholders to allow for knowledge co-creation, and integration of IK. After that, research findings on medicinal plants should be translated into user-friendly formats so that all stakeholders can easily locate and understand the findings. This would entail producing training guidelines, brochures, newsletters, policy briefs, seminars, media coverage and active community engagement.

*Training*: Stakeholders will need to be trained on effective and efficient ways to execute their roles within the value chain. Using available information, regular training workshops should be done to build and strengthen capacity. The focus should be on extension officers and community heads who are centrally positioned to support other farmers. Evaluations should be done based on changes in performance, based around the four main issues: institutional arrangements, leadership, knowledge, and accountability.

### 4.9 Key Stakeholders

To fully support the commercialisation of medicinal plants, actors or key stakeholders who directly and indirectly participate in the value chain need to be identified and their roles clearly stated. Table 2 identifies key experts and institutes that may be required for the successful commercialisation of medicinal plants and outlines their role.

## 5 Prioritisation of Functional Medicinal Plants

The report’s initial sections highlighted a wide range of NUFMS plants with potential within the nutraceutical and pharmaceutical industry. However, for allocating resources and developing targeted interventions, there is a need to streamline these and develop a priority list of NUFMS. The following key points should be noted:

1) The initial part of the report provided a list of NUFMS crops based on literature and was not exhaustive but provided a basis for prioritisation, and
2) Priority NUFMS should possess exceptional and desribale qualities (Supplementary Table S1) defined in terms of:
  a) *Nutritional value*: this is regarded as part of food quality and is a measure of a well-balanced ratio of the essential nutrients’ carbohydrates, fat, protein, minerals, and vitamins in items of food or diet concerning the nutrient requirements of their consumer,
  b) *Pharmaceutical value*: therapeutic indications, pharmacological effects, pharmacokinetic properties, physicochemical properties, and molecular mechanisms underlying the therapeutic benefits,
  c) *Cultural value:* forms part of the core principles and ideals upon which an entire community exists and protects and relies upon for existence and harmonious relationship*,*
  d) *Environmental importance*—provides several regulatory and support services to ecosystems which include, but are not limited to, photosynthesis, nutrient cycling, the creation of soils, and the water cycle, pollination, decomposition, water purification, erosion and flood control, and carbon storage and climate regulation, and
  e) *Economic potential:* the crop’s potential for economic development and growth and creation of surplus-value.

The list of priority NUFMS crops provided below is provisional. It still needs to be verified by relevant stakeholders such as traditional healers/herbalists, pharmaceuticals and the targeted industry, as part of a broader transdisciplinary approach.

*Cyclopia spp* (Honeybush tea) Currently, approximately 200 ha, mostly of *C. genistoides* and *C. subternata* shrubs, are under cultivation but cater to demand, wild harvesting, especially of *C. intermedia*, still contributes the major part of the annual production. It is estimated that 75% of honeybush tea is still harvested from the wild. Traditionally, leafy shoots and flowers were fermented and dried to prepare tea (Joubert et al., 2011). The increase in demand has placed natural populations not well-protected in jeopardy through unsustainable. Researchers have played a vital role in the “rediscovery” of this product and the development of the industry. Commercial cultivation and factory-based production have increased the access and value of honeybush tea. Extracts of the tea are gaining more scientific attention due to their phenolic composition (Agapouda et al., 2020).

*Athrixia phylicoides* DC. (Bush tea) is an indigenous South Africa shrub commonly used as an anti-depressant and aphrodisiac (Ajao et al., 2019). This shrub naturally grows in semi-arid regions with limited canopy coverage, such as grassland and forest biomes of South Africa (Limpopo, Free State, KwaZulu Natal and Eastern Cape Provinces) and Swaziland (Herman et al., 2000). Traditionally, the herb is greatly treasured as a traditional herbal medicine to treat diabetes, heart disease and hypertension, acne, boils, colds, cuts, headache, infected wounds, loss of voice and throat infection (Nchabeleng et al., 2013; Mathivha et al., 2019). It is also recommended as a potent blood cleanser (Mudau, 2012) and is further substantiated by its high levels of total polyphenol content (Mudau et al., 2005). The commercialisation of bush tea is a potential prospect in developing high-value products for the beverage and pharmaceutical industries (Tshivhandekano et al., 2018). There is, however, a need for an alternative supply of plant material as wild plants are under extreme pressure due to increased demands from local and export markets. Lerotholi et al. (2017) reported that the intensive harvesting of bush tea due to the increasing demand has, in many places, resulted in overexploitation and is a serious threat to biodiversity in the region. The only option for the crop species is sustainable conservation and cultivation to mitigate the impacts of unsustainable harvesting from the wild (Cunningham, 1993).

*Brassica oleracea* var. acephala (Kale) is a cruciferous vegetable characterised by leaves along the stem, which, in recent years, have gained great popularity as a “superfood”. Consequently, it is listed in many lists of the healthiest vegetables in popular culture. Although kale has been cultivated for several centuries and has been included in many traditional meals, especially in the Mediterranean, it has become very popular in South Africa. However, its popularity is as dichotomous as its households’ current food and nutrition security status. Kale is a superfood among the health-conscious upper-class, who primarily use it as an antioxidant booster. In the poor communities of SA, kale and its timely indigenisation and increased use support the notion of food crop globalisation (Traill, 1997; Ukonu, 2016; Liu et al., 2019). This notion has been supported by the southward migration of people from Africa’s central and western regions, where these leafy vegetables are considered a staple, to SA in search of employment opportunities (NEPAD (New Partnership for Africa’s Development, 2018). These crops have become indigenised and remain underutilised, creating opportunities to develop new value and transformation of those already existing, supporting rural agricultural development and food and nutrition security (Mabhaudhi et al., 2019). Kale has become a very popular crop among organic farmers due to its good tolerance for wide and extreme temperature fluctuations.

*Colocasia esculenta* (Taro) is a herbaceous perennial herb that grows to a height of 1–2 m. The main stem is an edible starch-rich underground structure (Chivenge et al., 2015). It is called the corm, from which leaves grow upwards, roots grow downwards while corms, cormels and runners grow laterally (Son et al., 2021). The root system is fibrous and confined mainly to the top layer of the soil (Sunitha et al., 2013). Corms in the dasheen type of taro are cylindrical and large. They are up to 30 cm long and 15 cm in diameter and constitute the main edible part of the plant (Modi and Mabhaudhi, 2013). In the eddboe types, the corms are small, globoid and surrounded by several corms and cormels. The corm and cormels constitute a significant portion of the edible harvest of eddoe taro. Since taro is gluten free and has low protein, high-calorie content, and low-fat levels, taro consumption can benefit individuals with dietary restrictions such as those presenting allergies, especially in children and gluten-intolerant individuals, contributing to reducing the risk of obesity and type II diabetes.

*Moringa oleifera* is a common tree native to India and cultivated throughout subtropical areas from West Africa to Fiji and is a source of food and medicine (Sreelatha and Padma, 2011). In South Africa, moringa grows in the Limpopo, Free State, Mpumalanga, KwaZulu-Natal and Gauteng provinces. According to traditional African and Indian (Ayurvedic) medicine, moringa has almost 540 compounds that can treat or prevent 300 health issues (Freiberger et al., 1998; Rivas et al., 2013; Aderinola et al., 2019). In developing countries, the moringa leaf powder is commonly used as a medicinal herb rather than food, as in Asian populations. It is often taken as a supplement by HIV-infected people to enhance immunity and manage opportunistic infections. In SA, several flagship projects have been initiated due to growing interest. One example includes the Agricultural Research Council’s Vegetable and Ornamental Plants group in Roodeplaat researching moringa propagation, cultivation practices, processing, storage (shelf-life) and analysis on biological activities, safety and phytochemistry (Mashamaite et al., 2021).

## 6 Conclusion

Several neglected and underutilised crops have nutraceutical and pharmaceutical properties that make them ideal for commercialisation. Also, they can contribute to sustainable and healthy diets and sustainable agricultural intensification, making them an important resource for food systems transformation. Thus, they can deliver on multiple objectives. Currently, unsustainable harvesting and utilisation of wild populations threatens several species, which are now endangered and facing extinction. Sustainable conservation and cultivation practices are urgently needed to mitigate further population declines in the wild. Their nutraceutical and pharmaceutical properties provide a clear starting point for developing commercial food and herbal medicinal products. To effectively meet the requirements for nutraceutical and pharmaceutical development, there is a need to develop their value chains; however, limited research and development done so far limit their potential. Therefore, RDI should focus on all aspects of their value chain to unlock their commercial potential and develop new inclusive value chains. Transdisciplinary approaches are needed to ensure that these new value chains are well-supported by public and private sectors, inclusive and equitable.

## Data Availability

The original contributions presented in the study are included in the article/Supplementary Material, further inquiries can be directed to the corresponding authors.
