# Supplementary material for: Neglected and Underutilised Crops: A Systematic Review of Their Potential as Food and Herbal Medicinal Crops in South Africa
Source: Front Pharmacol. 2022 Jan 20;12:809866. doi: 10.3389/fphar.2021.809866 (PMC8811033; doi:10.3389/fphar.2021.809866)
Supplement: Supplementary file 1 [file Table1.docx]

**Supplementary Table 1**: Search scope for Nutraceutical and pharmaceutical properties of neglected and underutilised crops

| Query string | CODE | HITS | |  |
| --- | --- | --- | --- | --- |
| Title, Abstract, Keywords |  | SCOPUS | WOS | PubMed |
| The terms nutraceutical OR pharmacological |  |  |  |  |
| (nutraceutical OR pharmacological OR phytochemical OR pharmaceutical or medicinal) | #1 | 801 235 | 573 011 | 419 998 |
| Nutraceutical and pharmacological characteristics |  |  |  |  |
| ("Anti- "AND (oxidant OR fungal OR bacterial OR viral OR mutagenic OR hepatoxic OR inflammatory OR histaminic OR immunomodulatory OR hypolipidemic OR diabetic OR convulsant OR carcinogenic OR hypolipidemic OR acetylcholinesterase OR neuropathic OR hypertensive OR analgesia OR malaria)  OR  (Lactogenic OR aphrodisiac OR diuretic OR hepatoprotective OR hypotensive OR carminative)) | #2 | 805 921 | 337 149 | 466 116 |
| (Antifungal OR antibacterial OR antiviral OR antimutagenic OR Antihepatoxic OR Anti-inflammatory OR Antihistaminic OR antiimmuno-modulatory OR antihypolipidemic OR antidiabetic OR anticonvulsant OR anticarcinogenic OR antihypolipidemic OR antiacetylcholinesterase OR antineuropathic OR antihypertensive OR antianalgesic OR lactogenic OR aphrodisiac OR diuretic OR hepatoprotective OR hypotensive OR carminative) | #3 | 958 943 | 527 836 | 547 661 |
| (Anti-fungal OR anti-bacterial OR anti-viral OR anti-mutagenic OR Anti-hepatoxic OR Anti-inflammatory OR Anti-histaminic OR anti-immuno-modulatory OR anti-hypolipidemic OR anti-diabetic OR anti-convulsant OR anti-carcinogenic OR anti-hypolipidemic OR anti-acetylcholinesterase OR anti-neuropathic OR anti-hypertensive OR anti-analgesic OR lactogenic OR aphrodisiac OR diuretic OR hepatoprotective OR hypotensive OR carminative) | #4 | 578 390 | 272 043 | 248 294 |
| Nutraceutical and Pharmaceutical characteristics [#2 OR #3 OR #4] | #5 | 248 028 | 822 204 | 752 433 |
| Neglected and underutilised crops |  |  |  |  |
| (indigenous OR neglected OR traditional OR orphan OR native OR underutili?ed OR future OR medicinal) PRE/2 crop*) | #6 | 5 222 | 3 998 | 406 |
| Nutraceutical And pharmaceutical properties of NUS |  |  |  |  |
| To address RQ1 -The mention of Nutraceutical and Pharmaceutical properties in NUS [#1 AND #6] | #7 | 361 | 364 | 60 |
| To address RQ2 -Nutraceutical and Pharmaceutical characteristics in NUS [#5 AND #6] | #8 | 78 | 52 | 6 |
| Combined search [#7 OR #8] | #9 | 384 | 383 | 62 |
| Remove duplicates | 339 | | | |
| Removal of articles that were not in English | 329 | | | |
| Removal of articles where full texts could not be downloaded | 223 | | | |
| Removal of irrelevant articles (no explicit mention of Nutraceutical and Pharmaceutical properties of an underutilised crop) | 106 | | | |
| Total | 106 | | | |
