# Supplementary material for: Neglected and Underutilised Crops: A Systematic Review of Their Potential as Food and Herbal Medicinal Crops in South Africa
Source: Front Pharmacol. 2022 Jan 20;12:809866. doi: 10.3389/fphar.2021.809866 (PMC8811033; doi:10.3389/fphar.2021.809866)
Supplement: Supplementary file 2 [file Table2.docx]

Supplementary Table 2: Scoring of pharmaceutical (P), nutraceutical (N), cultural (C), environmental (En) and economic (Ec) potential for neglected and underutilised functional medicinal crop species. The scores used were (*) low, (**) medium and (***) high

| **Common name** | **Scientific name** | **P^1^** | **N** | **C** | **En** | **Ec** |
| --- | --- | --- | --- | --- | --- | --- |
| Honeybush | *Cyclopia* (Vent.) *spp* | *** | *** | *** | *** | *** |
| Bush tea | *Athrixia phylicoides* DC. | *** | *** | *** | *** | *** |
| Tigernut | *Cyperus esculentus* (L.) | *** | ** | * | * | *** |
| Ground Bean | *Macrotyloma geocarpum* (Harms) Maréchal & Baudet | ** | *** | * | * | ** |
| Winged bean | *Psophocarpus tetragonolobus* (L.) D.C. | ** | ** | * | ** | * |
| Sword bean | *Canavalia gladiate* (Jacq.) DC. | ** | ** | * | ** | * |
| Sunn hemp | *Crotalaria juncea*  (L.) | *** | ** | * | ** | ** |
| Lablab | *Dolichos lablab* (L.) *or Lablab purpureus*  (L.) Sweet | *** | ** | * | ** | *** |
| Pigeon pea | *Cajanus cajan* (L.) Millsp. | *** | ** | * | ** | *** |
| Winged bean | *Psophocarpus tetragonolobus* (L.) D.C. | ** | ** | ** | ** | ** |
| Bambara groundnut | *Vigna subterranea* (L.) Verdc. | ** | *** | ** | ** | *** |
| Velvet bean | *Mucuna pruriens* (L.) DC. var utilis | ** | ** | * | ** | * |
| Grass pea | *Lathyrus sativus* (L.) | ** | *** | * | * | * |
| Clusterbean | *Cyamopsis tetragonoloba* (L.) Taub. | ** | ** | * | * | * |
| Broad bean | *Vicia faba* (L.) | *** | ** | * | ** | ** |
| African yam bean | *Sphenostylis stenocarpa* (Hochst. ex A.Rich.) Harms | ** | ** | * | ** | *** |
| Black gram | *Vigna mungo* (L.) Hepper | ** | ** | * | * | * |
| Drumstick | *Moringa oleifera* (L.) | *** | *** | *** | *** | *** |
| Baobab/ African Baobab | *Adansonia digitate* (L.) | *** | *** | *** | ** | ** |
| Breadfruit | *Artocarpus altilis* (Parkinson) Fosberg | ** | *** | * | ** | * |
| African fan palm | *Borassus aethiopum* (Mart.) | ** | * | * | * | * |
| Blackberry | *Rubus fruticosus* (L.) | ** | *** | * | * | * |
| Mulberry | *Morus alba* (L.) | ** | ** | ** | ** | * |
| Physic nut | *Jatropha curcas* (L.) | *** | *** | * | * | * |
| Jackfruit | *Artocarpus heterophyllus* (Lam.) | * | ** | * | *** | * |
| Velvet tamarind | *Dialium guineense* (Willd.) | *** | * | * | * | * |
| Marvel of Peru or four o'clock flower | *Mirabilis jalapa* (L.) | ** | ** | * | ** | * |
| Chocolate weed | *Melochia corchorifolia* (L.) | *** | * | ** | ** | *** |
| Cannabis | *Cannabis sativa* (L.) | ** | *** | ** | ** | *** |
| Hibiscus or Roselle | *Hibiscus sabdariffa* (L.) | ** | ** | * | ** | ** |
| Cape periwinkle; graveyard plant | *Catharanthus roseus* (L.) G.Don | ** | ** | * | *** | * |
| Cassava | *Manihot esculenta* (Crantz) | ** | ** | * | ** | ** |
| Donkey berry | *Grewia flavescens* (Juss) | ** | ** | ** | ** | *** |
| Carob | *Ceratonia silique* (L.) | ** | ** | * | ** | ** |
| Cancer bush | *Sutherlandia frutescens* (L.) R.Br. *or*  *Lessertia frutescens* (L.) Goldblatt & J.C.Manning | ** | ** | ** | ** | ** |
| Ethiopian eggplant | *Solanum aethiopicum* (L.) | ** | ** | * | ** | ** |
| African eggplant | *Solanum macrocarpon* (L.) | ** | ** | * | ** | * |
| Bitter eggplant | *Solatium insanum,* (L.) | ** | ** | ** | ** | *** |
| Miracle fruit | *Synsepalum dulcificum* (Schumach. & Thonn.) William Freeman Danielfer | ** | ** | * | ** | * |
| Cactus pear | *Opuntia robusta* J.C.Wendl. ex Pfeiff. | ** | ** | ** | ** | *** |
| Pea eggplant | *Solanum torvum* (Sw.) | * | ** | ** | ** | *** |
| Quinoa | *Chenopodium quinoa* C.L. Willdenow (Willd.) | ** | ** | *** | ** | *** |
| Buck wheat | *Fagopyrum esculentum* (Moench) | ** | *** | *** | ** | *** |
| Amaranth | *Amaranthus spinosus* (L.) | ** | ** | * | ** | * |
| Amaranth | *Amaranthus tricolor* (L.) | ** | ** | * | ** | * |
| Elephant foot yam | *Amorphophalus campanulatus* (Dennst.) Nicolson | ** | ** | * | ** | * |
| Up yam | *Dioscorea bulbiferaa* (L.) | ** | ** | * | ** | * |
| Lesser yam | *Dioscorea esculenta* (Lour.) Burkill | *** | *** | ** | *** | ** |
| Taro | *Colocasia esculenta* (L.) Schott | ** | *** | *** | *** | * |
| Greater yam | *Dioscorea alata* (L.) | ** | ** | * | ** | * |
| Yam | *Dioscorea dumetorum* (Kunth) Pax | ** | *** | ** | ** | ** |
| Sweet potato | *Ipomea batatas* (L.) Lam. | ** | *** | ** | *** | * |
| Giant taro | *Alocasia macrorrhiza*(L.) G.Don | ** | *** | ** | ** | ** |
| Ethiopian potato | *Plectranthus edulis* (Vatke) A.J.Paton | ** | *** | ** | ** | * |
| Wild ginger | *Siphonochilus aethiopicus* (Schweinf.) B.L.Burtt | ** | *** | ** | ** | *** |
| Tannia | *Xanthosoma sagittifolium* (L.) Schott | ** | *** | ** | ** | * |
| White seed melon | *Cucumeropsis manni* (Naudin) | ** | *** | ** | ** | * |
| Watermelon | *Citrullus lanatus* (Thunb.) Matsum. & Nakai | ** | *** | ** | ** | * |
| Bottle gourd | *Lagenaria siceraria* (Molina) Standl. | ** | *** | ** | ** | * |
| Bitter gourd | *Momordica charantia* (L.) | ** | *** | ** | *** | *** |
| Wax gourd | *Benincasa hispida* ((Thunb.) Cogn.) | ** | *** | *** | ** | ** |
| Bitter melon | *Momordica charantia* (L.) | ** | *** | ** | ** | * |
| Pumpkin | *Cucurbita pepo var. styriaca* (L.) | * | *** | *** | ** | ** |
| Napier grass | *Pennisetum purpureum* (Schumach) | * | *** | *** | ** | ** |
| Pearl millet | *Cenchrus americanus* (L.) Morrone | * | *** | ** | ** | * |
| Barley | *Hordeum vulgare (*L.) | * | *** | ** | ** | * |
| Proso millet | *Panicum miliaceum* (L.) | * | *** | ** | ** | * |
| Fonio millet | *Digitaria exilis* (Kippist) Stapf | * | *** | ** | ** | * |
| Foxtail millet | *Setaria italica* (L.) P. Beauvois | ** | ** | *** | ** | *** |
| Finger millet | *Eleusine coracana* (L.) | ** | *** | ** | ** | * |
| Sorghum | *Sorghum bicolor* (L.) | ** | ** | *** | ** | *** |
| Maize | *Zea mays* (L.) | ** | ** | *** | ** | *** |
| Scarlet pimpernel | *Chenopodium album* (L.) | ** | ** | *** | ** | *** |
| Sweet clover | *Melilotus officinalis* (L.) Pall. | ** | *** | * | ** | ** |
| Lemongrass | *Cymbopogon flexuosus* (Nees ex Steud.) W.Watson | ** | *** | ** | ** | * |
| Rapeseed/ Sarson | *Brassica napus* (L.) | ** | *** | ** | ** | *** |
| Water hyssop | *Bacopa monnieri* (L.) Pennell | ** | *** | ** | ** | *** |
| Safflower | *Carthamus tinctorius* (L.) | ** | *** | *** | ** | ** |
| Fennel flower | *Nigella sativa* (L.) | ** | *** | * | ** | * |
| Ladies' fingers or Okra | *Abelmoschus esculentus* (L.) Moench | ** | *** | * | ** | * |
| Plantain | *Plantago major* (L.) | ** | *** | * | ** | * |
| Toothache plant | *Acmella oleracea* (L.) R.K.Jansen | ** | *** | * | *** | * |
| Creeping woodsorrel | *Oxalis corniculata* (L.) | ** | *** | *** | *** | * |
| Chinese water chestnut | *Eleocharis dulcis* (Burm.f.) Trin. ex Hensch. | ** | ** | * | *** | * |
| Lamb’s quarters | *Chenopodium album (*L.) | ** | ** | *** | *** | ** |
| False sesame | *Ceratotheca sesamoides* (Endl.) | ** | ** | *** | *** | ** |
| Black sesame | *Sesamum radiatum* (Schumach. and Thonn) | ** | ** | *** | *** | ** |
|  | *Crassocephalum rubens* (Juss. and Jacq.) S. | * | ** | *** | *** | * |
| Spiderflower | *Cleome gynandra* (L.) | ** | ** | ** | *** | * |
| Jute mallow | *Corchorus olitorius* (L.) | ** | ** | ** | *** | * |
| Thickhead, redflower ragleaf | *Crassocephalum crepidioides* (Benth.) S.Moore | ** | ** | *** | *** | * |
| Wild mustard | *Brassica juncea* (L.) | ** | ** | ** | *** | * |
| Kales | *Brassica oleracea* (L.) | *** | *** | *** | ** | *** |
| Common dandelion | *Taraxacum officinale* F. H. Wigg | *** | ** | *** | ** | * |
| Purslane | *Portulaca oleracea* (L.) | ** | ** | ** | ** | * |
| Hyacinthus | *Hyacinthaceae* | ** | ** | ** | ** | * |
