# Supplementary material for: Neglected and Underutilised Crops: A Systematic Review of Their Potential as Food and Herbal Medicinal Crops in South Africa
Source: Front Pharmacol. 2022 Jan 20;12:809866. doi: 10.3389/fphar.2021.809866 (PMC8811033; doi:10.3389/fphar.2021.809866)
Supplement: Supplementary file 3 [file Table3.docx]

**Supplementary Table 3: Clustering of keywords identified in Vosviewer on NUFMS**

| **Colour** | **Keywords** | **Cluster** |
| --- | --- | --- |
| Red | Amaranth | 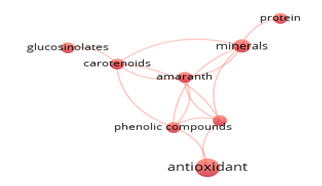 |
|  | Antioxidant |  |
|  | Carotenoids |  |
|  | Glucosinolates |  |
|  | Minerals |  |
|  | Phenolic compounds |  |
|  | Protein |  |
|  | Purslane |  |
| Green | Buckwheat | 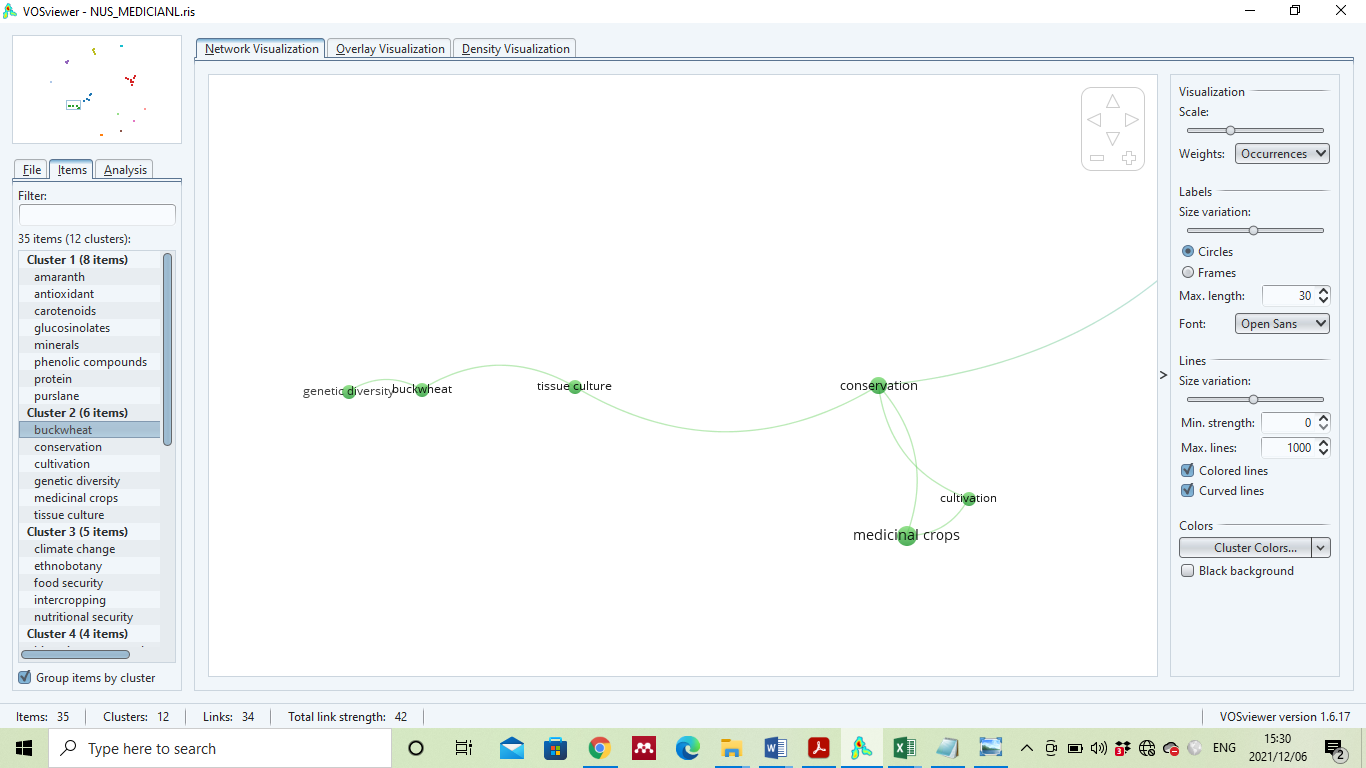 |
|  | Conservation |  |
|  | Cultivation |  |
|  | Genetic diversity |  |
|  | Medicinal crops |  |
|  | Tissue culture |  |
| Blue | Climate change | 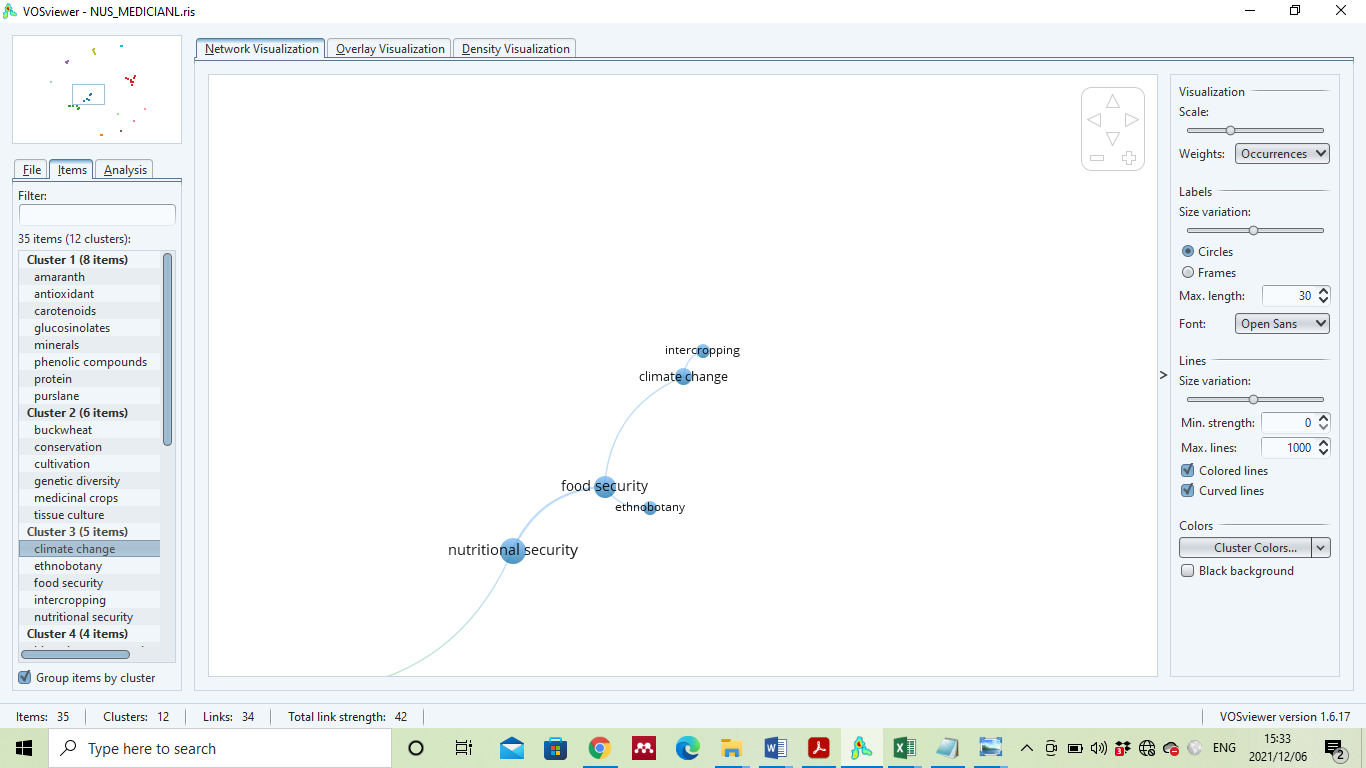 |
|  | Ethnobotany |  |
|  | Food security |  |
|  | Intercropping |  |
|  | Nutritional security |  |
| Lime | Bioactive compounds | 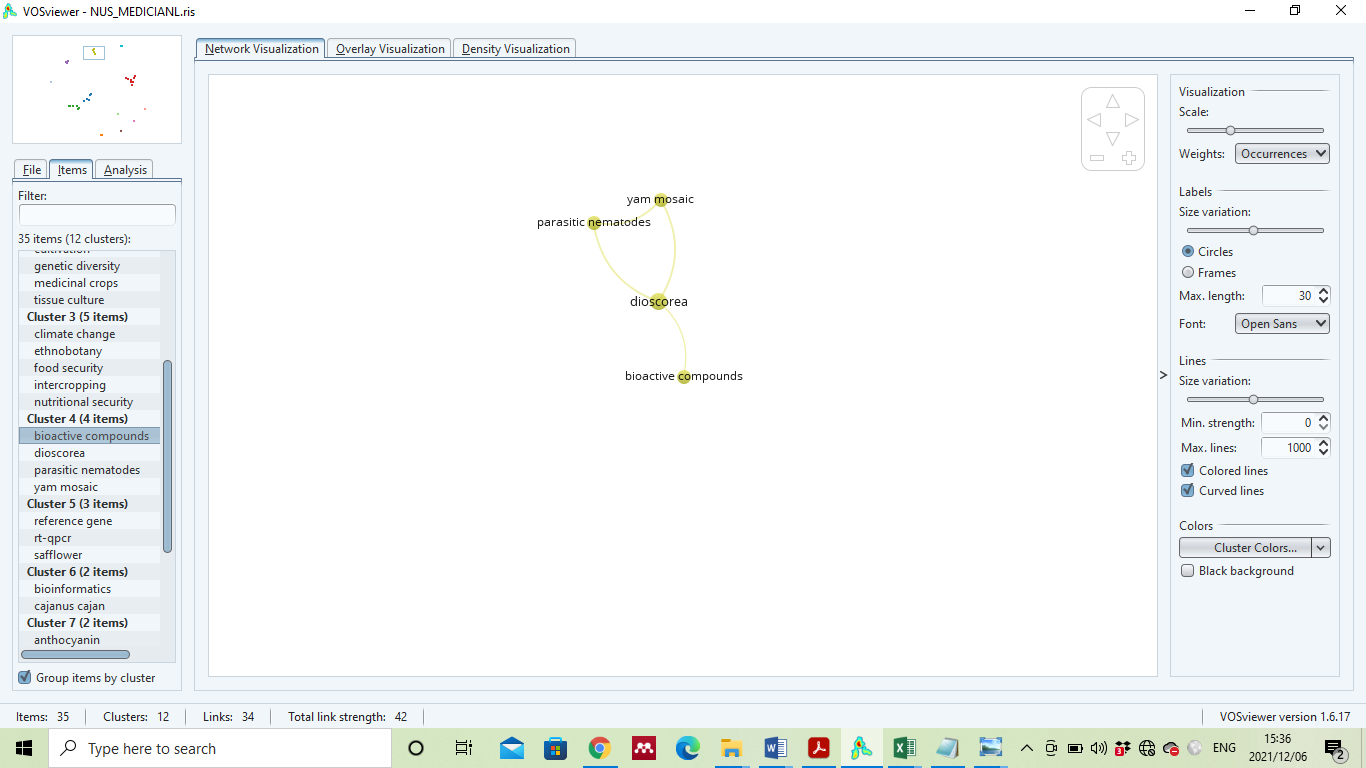 |
|  | Dioscorea |  |
|  | Parasitic nematodes |  |
|  | Yam mosaic |  |
| Purple | Reference gene | 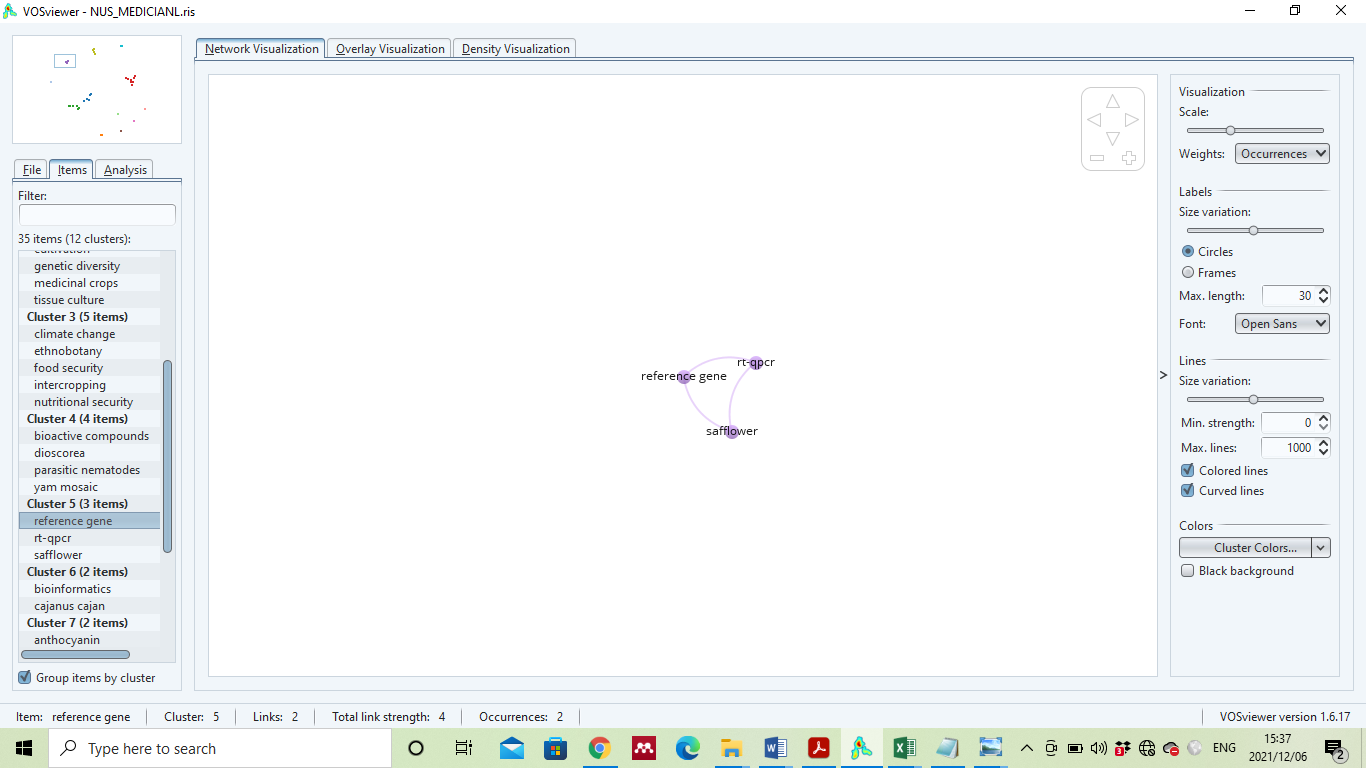 |
|  | Rt-qpcr |  |
|  | Safflower |  |
| Sky blue | Bioinformatics | 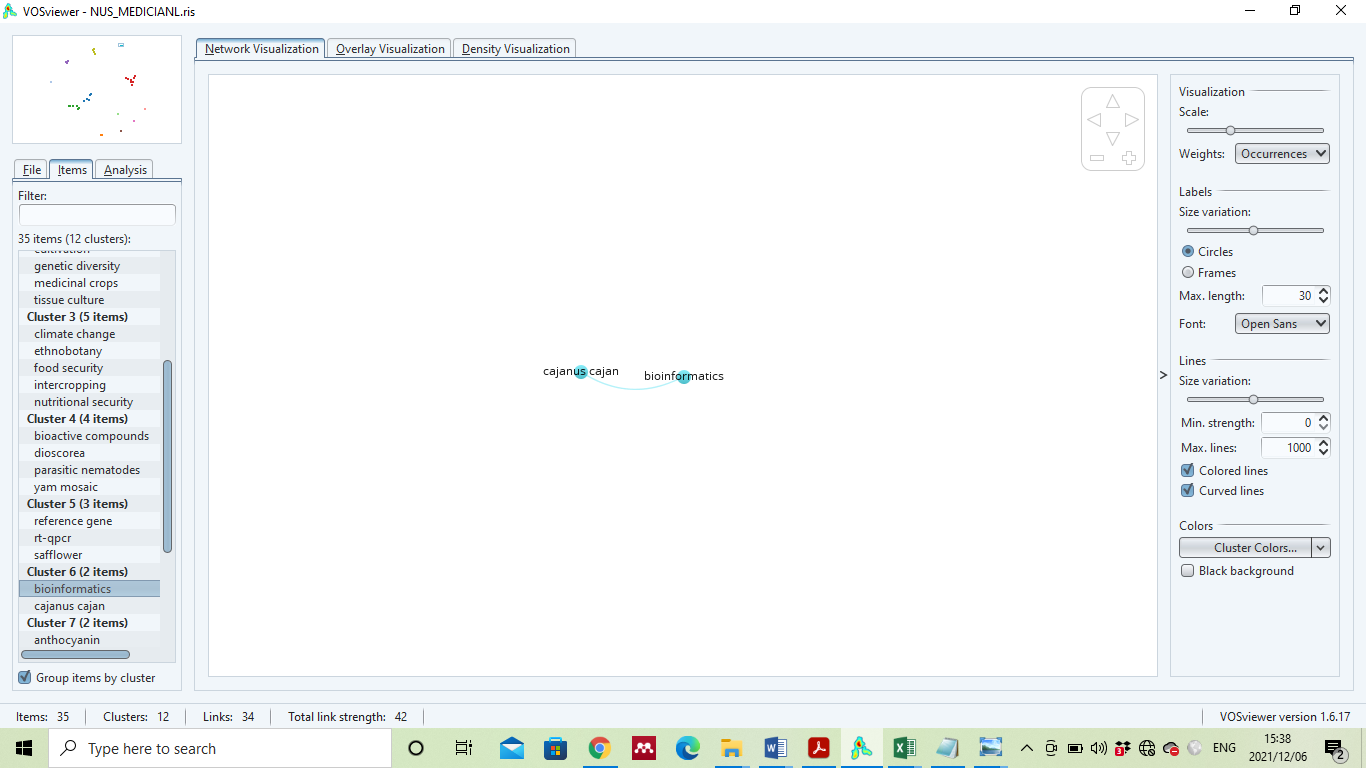 |
|  | Cajanus cajan |  |
| Orange | Anthocyanin | 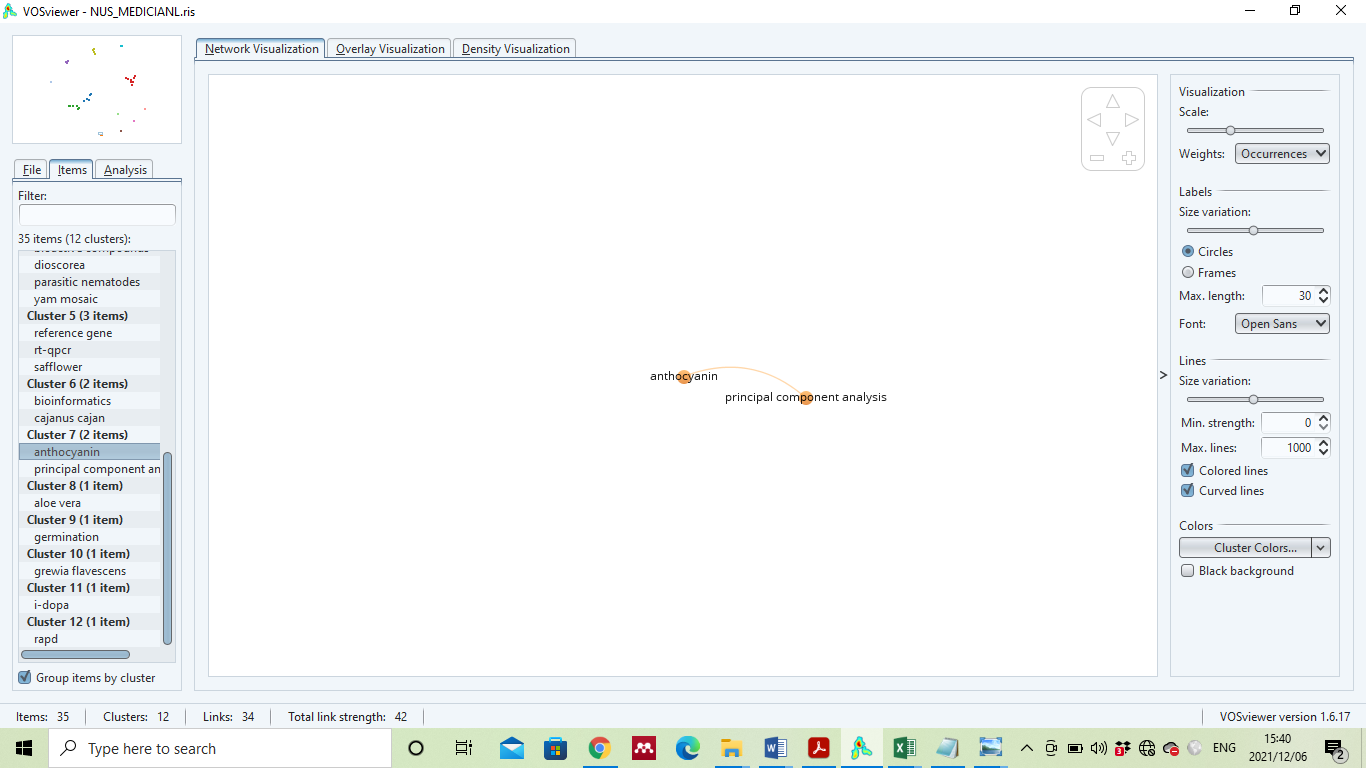 |
|  | Principal component analysis |  |
| Brown | Aloe vera | 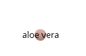 |
| Lilac | Germination | 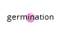 |
| Peach | Grewia flavescens | 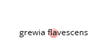 |
| Light green | i-dopa | 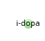 |
| Navy blue | Rapd | 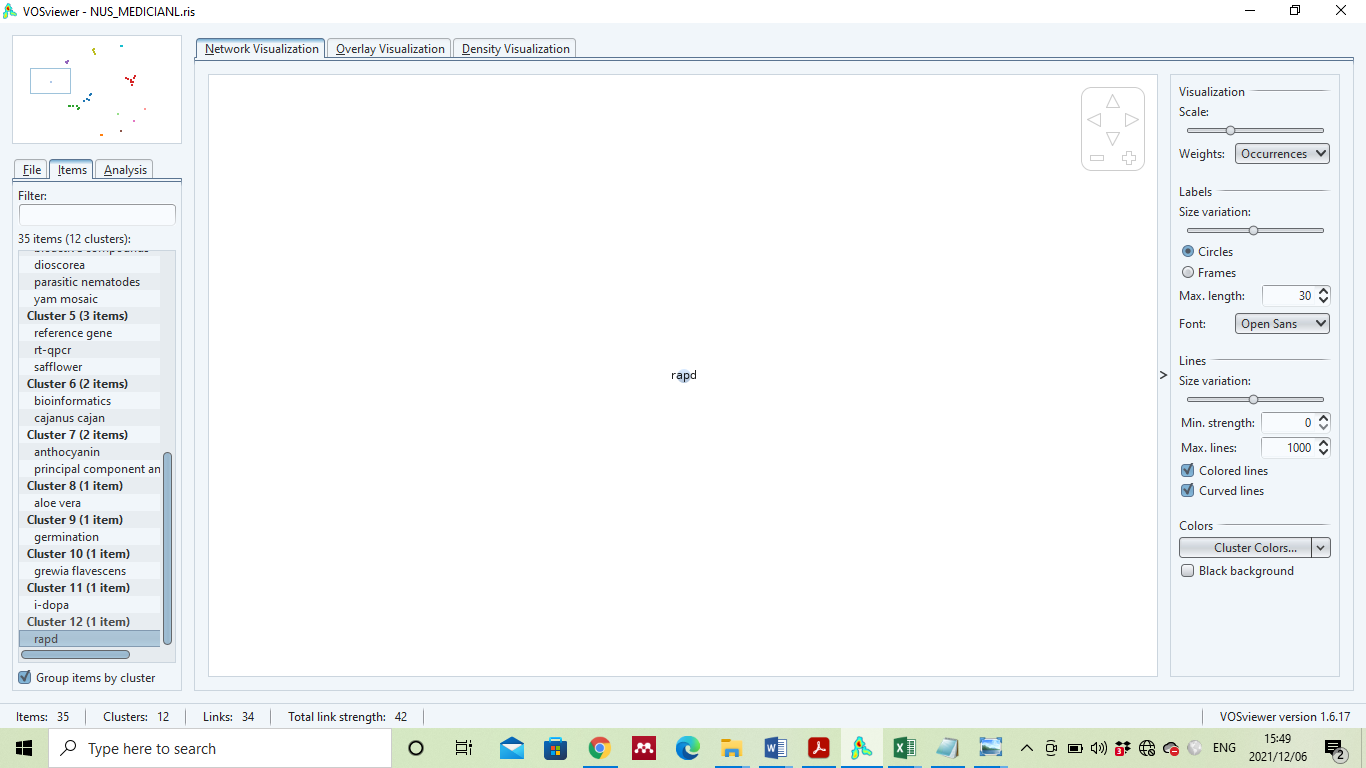 |
