# Supplementary material for: Neglected and Underutilised Crops: A Systematic Review of Their Potential as Food and Herbal Medicinal Crops in South Africa
Source: Front Pharmacol. 2022 Jan 20;12:809866. doi: 10.3389/fphar.2021.809866 (PMC8811033; doi:10.3389/fphar.2021.809866)
Supplement: Supplementary file 5 [file DataSheet1.docx]

Supplementary information: Reference list of articles used to construct Table 1

Achten, W.M.J., Maes, W.H., Reubens, B., Mathijs, E., Singh, V.P., Verchot, L., Muys, B., 2010. Biomass production and allocation in Jatropha curcas L. seedlings under different levels of drought stress. Biomass and Bioenergy 34, 667–676. doi:10.1016/j.biombioe.2010.01.010

Aderinola, T.A., Fagbemi, T.N., Enujiugha, V.N., Alashi, A.M., Aluko, R.E., 2019. In vitro antihypertensive and antioxidative properties of alcalase-derived Moringa oleifera seed globulin hydrolysate and its membrane fractions. J. Food Process. Preserv. 43. doi:10.1111/jfpp.13862

Al-Sayed, H.M., Hegab, S.A., Youssef, M.A., Khalafalla, M.Y., Almaroai, Y.A., Ding, Z., Eissa, M.A., 2020. Evaluation of quality and growth of roselle (Hibiscus sabdariffa L.) as affected by bio-fertilizers. J. Plant Nutr. 43, 1025–1035. doi:10.1080/01904167.2020.1711938

Amjad, M., Akhtar, S.S., Yang, A., Akhtar, J., Jacobsen, S.E., 2015. Antioxidative Response of Quinoa Exposed to Iso-Osmotic, Ionic and Non-Ionic Salt Stress. J. Agron. Crop Sci. 201, 452–460. doi:10.1111/jac.12140

Avashthi, H., Pathak, R.K., Pandey, N., Arora, S., Mishra, A.K., Gupta, V.K., Ramteke, P.W., Kumar, A., 2018. Transcriptome-wide identification of genes involved in Ascorbate–Glutathione cycle (Halliwell–Asada pathway) and related pathway for elucidating its role in antioxidative potential in finger millet (Eleusine coracana (L.)). 3 Biotech 8. doi:10.1007/s13205-018-1511-9

Bashir, E., Mahajan, R., Mir, R.A., Dar, W.A., Zargar, S.M., 2021. Unravelling the genetic variability and population structure of buckwheat (Fagopyrum spp.): a collection of north western Himalayas. Nucl. 64, 93–101. doi:10.1007/s13237-020-00319-y

Batlle, I., Tous, J., 1997. Carob tree. Ceratonia siliqua L. Promoting the conservation and use of underutilized and neglected crops, Institute of Plant Genetics and Crop Plant Research, Gatersleben/International Plant Genetic Resources Institute, Rome. Italy.

Bedi, E.N., Peter-ikechukwu, A.I., 2015. Comparison of Proximate Composition of Some Cultivars of Chickpea ( Cicer arietinum L .) Cultivated in Owerri , Imo State , Nigeria 37, 103–110.

Bhattacharjee, T., Sen, D., Irenaeus, T.K.S., Das, S.P., Chakraborty, A., 2019. Diversity of underutilized vegetable crops in Tripura. Acta Hortic. 1241, 121–134. doi:10.17660/ActaHortic.2019.1241.19

Bolade, M.K., Bello, S.B., 2006. Selected physicochemical properties of flour from the root of african fan palm (borassus aethiopum). Int. J. Food Prop. 9, 701–713. doi:10.1080/10942910600575641

Bungla, P.S., Tewari, G., Rawal, R.S., Bhatt, I.D., Tewari, L.M., Jugran, A.K.R., Mohan, B., 2017. Comparative in vitro antioxidant activity, morphological and phytochemical profiling of traditional crops from Kumaim Himalaya. J. Indian Chem. Soc. 94, 181–189.

Caplan, D., Dixon, M., Zheng, Y., 2019. Increasing inflorescence dry weight and cannabinoid content in medical cannabis using controlled drought stress. HortScience 54, 964–969. doi:10.21273/HORTSCI13510-18

Ceballos, A., Valdivia, C., Rivera, J., Arce, M., 2012. Velvet Bean (Mucuna pruriens var. utilis) a cover crop as Bioherbicide to preserve the environmental services of soil.

Chalwe, A., Chiona, M., Simwambana, M.S.C., 2002. Improving Household Food Security by using Planted Fallows in Zambia. Discov. Innov. 14, 76–81.

Chay, S.Y., Salleh, A., Sulaiman, N.F., Zainal Abidin, N., Hanafi, M.A., Zarei, M., Saari, N., 2018. Blood-pressure lowering efficacy of winged bean seed hydrolysate in spontaneously hypertensive rats, peptide characterization and a toxicity study in Sprague-Dawley rats. Food Funct. 9, 1657–1671. doi:10.1039/c7fo01769c

Chibarabada, T.P., Modi, A.T., Mabhaudhi, T., 2017. Nutrient content and nutritional water productivity of selected grain legumes in response to production environment. Int. J. Environ. Res. Public Health 14, 1300. doi:10.3390/ijerph14111300

Chung, I.M., Thiruvengadam, M., Rekha, K., Rajakumar, G., 2016. Elicitation Enhanced the Production of Phenolic Compounds and Biological Activities in Hairy Root Cultures of Bitter melon (Momordica charantia L.). Brazilian Arch. Biol. Technol. 59, 1–10. doi:10.1590/1678-4324-2016160393

Crop, A., Society, S., 2019. Positive Selection and Neem Leaf Powder Factors 27, 631–640.

Dagar, J.C., Tomar, O.S., Minhas, P.S., Kumar, M., 2013. Lemongrass (Cymbopogon flexuosus) productivity as affected by salinity of irrigation water, planting method and fertilizer doses on degraded calcareous soil in a semi-arid region of northwest India. Indian J. Agric. Sci. 83, 734–738.

Dansi, a., Vodouhè, R., Azokpota, P., Yedomonhan, H., Assogba, P., Adjatin, a., Loko, Y.L., Dossou-Aminon, I., Akpagana, K., 2012. Diversity of the neglected and underutilized crop species of importance in benin. Sci. World J. 2012, 1–19. doi:10.1100/2012/932947

De Caluwé, E., De Smedt, S., Assogbadjo, A.E., Samson, R., Sinsin, B., Van Damme, P., 2009. Ethnic differences in use value and use patterns of baobab (Adansonia digitata L.) in northern Benin. Afr. J. Ecol. 47, 433–440. doi:10.1111/j.1365-2028.2008.01023.x

Fandohan, B., Assogbadjo, A.E., Kakaï, R.G., Kyndt, T., de Caluwé, E., Codjia, J.T.C., Sinsin, B., 2010. Women’s Traditional Knowledge, Use Value, and the Contribution of Tamarind (Tamarindus indica L.) to Rural Households’ Cash Income in Benin. Econ. Bot. 64, 248–259. doi:10.1007/s12231-010-9123-2

Galvao, A.C., Nicoletto, C., Zanin, G., Vargas, P.F., Sambo, P., 2021. Nutraceutical content and daily value contribution of sweet potato accessions for the European market. Horticulturae 7, 1–14. doi:10.3390/horticulturae7020023

Gangadhar, L., Praseetha, P.K., 2019. Identification and molecular phylogenetic relationship of selected medicinal plants-ethano medicinal importance. Indian J. Public Heal. Res. Dev. 10, 1248–1252. doi:10.5958/0976-5506.2019.01842.4

Gebauer, J., Bernholt, H., Hammer, K., 2013. Grewia flavescens: A potential horticultural crop? Genet. Resour. Crop Evol. 60, 1915–1919. doi:10.1007/s10722-013-0003-3

Geldenhuys, C.J., 2007. Weeds or useful medicinal plants in the rural home garden? Food Nutr. Bull. 28, S392–S397. doi:10.1177/15648265070282s219

Giambanelli, E., Verkerk, R., D’Antuono, L.F., Oliviero, T., 2016. The kinetic of key phytochemical compounds of non-heading and heading leafy Brassica oleracea landraces as affected by traditional cooking methods. J. Sci. Food Agric. 96, 4772–4784. doi:10.1002/jsfa.7844

Grimaldi, I.M., Leke, W.N., Borokini, I., Wanjama, D., Van Andel, T., 2018. From landraces to modern cultivars: field observations on taro Colocasia esculenta (L.) Schott in sub-Saharan Africa. Genet. Resour. Crop Evol. 65, 1809–1828. doi:10.1007/s10722-018-0651-4

Gulla, A., Getachew, A., Haile, T.G., Molla, F., 2020. Evaluation of Acid-Modified Ethiopian Potato (Plectranthus edulis) Starch as Directly Compressible Tablet Excipient. Biomed Res. Int. 2020. doi:10.1155/2020/9325173

Gullino, M.L., Gilardi, G., Bertetti, D., Garibaldi, A., 2020. Emerging soilborne pathogens and trends in their management. Acta Hortic. 1270, 9–21. doi:10.17660/ActaHortic.2020.1270.2

Hall, N.D., Patel, J.D., McElroy, J.S., Goertzen, L.R., 2021. Detection of subgenome bias using an anchored syntenic approach in Eleusine coracana (finger millet). BMC Genomics 22. doi:10.1186/s12864-021-07447-y

Hendawy, S., Abouziena, H., Abd El-Razik, T., Amer, H., Hussein, M., 2019. Winter weeds and its control in the medicinal plants in Egypt: a survey study. Egypt. Pharm. J. 18, 16. doi:10.4103/epj.epj_13_18

Horbowicz, M., Obendorf, R.L., 2005. Fagopyritol accumulation and germination of buckwheat seeds matured at 15, 22, and 30°C. Crop Sci. 45, 1264–1270. doi:10.2135/cropsci2004.0431

Janardhanan, V., 2000. Nutritional and anti-nutritional composition of velvet bean: an under-utilized food legume in south India. J. food Sci. Nutr.

Joubert, E., Gelderblom, W.C.A., Louw, A., de Beer, D., 2008. South African herbal teas: Aspalathus linearis, Cyclopia spp. and Athrixia phylicoides—A review. J. Ethnopharmacol. 119, 376–412. doi:10.1016/j.jep.2008.06.014

Joubert, E., Joubert, M.E., Bester, C., de Beer, D., De Lange, J.H., 2011. Honeybush (Cyclopia spp.): From local cottage industry to global markets — The catalytic and supporting role of research. South African J. Bot. 77, 887–907. doi:https://doi.org/10.1016/j.sajb.2011.05.014

Kaizzi, C.K., Ssali, H., Vlek, P.L.G., 2004. The potential of Velvet bean (Mucuna pruriens) and N fertilizers in maize production on contrasting soils and agro-ecological zones of East Uganda. Nutr. Cycl. Agroecosystems 68, 59–72. doi:10.1023/B:FRES.0000012233.27360.60

Kamble, D.B., Singh, R., Rani, S., Kaur, B.P., Upadhyay, A., Kumar, N., 2019. Optimization and characterization of antioxidant potential, in vitro protein digestion and structural attributes of microwave processed multigrain pasta. J. Food Process. Preserv. 43. doi:10.1111/jfpp.14125

Khan, Z.R., Midega, C.A.O., Bruce, T.J.A., Hooper, A.M., Pickett, J.A., 2010. Exploiting phytochemicals for developing a “push-pull” crop protection strategy for cereal farmers in Africa. J. Exp. Bot. 61, 4185–4196. doi:10.1093/jxb/erq229

Kleynhans, R., Bulannga, M., Nenungwi, L., Lehlaleroa, M.T., Matsiliza-Mlathi, B., Slabbert, M.M., 2018. Preliminary investigations on germination of Sutherlandia frutescens and emergence of Ceratonia siliqua seed. Acta Hortic. 1204, 153–159. doi:10.17660/ActaHortic.2018.1204.20

Koley, T.K., Khan, Z., Oulkar, D., Singh, B., Bhatt, B.P., Banerjee, K., 2020. Profiling of polyphenols in phalsa (Grewia asiatica L) fruits based on liquid chromatography high resolution mass spectrometry. J. Food Sci. Technol. 57, 606–616. doi:10.1007/s13197-019-04092-y

Kumari, A., Chaudhary, H.K., 2020. Nutraceutical crop buckwheat: a concealed wealth in the lap of Himalayas. Crit. Rev. Biotechnol. 40, 539–554. doi:10.1080/07388551.2020.1747387

Kushwaha, R., Fatima, N.T., Singh, M., Singh, V., Kaur, S., Puranik, V., Kumar, R., Kaur, D., 2021. Effect of cultivar and maturity on functional properties, low molecular weight carbohydrate, and antioxidant activity of Jackfruit seed flour. J. Food Process. Preserv. 45. doi:10.1111/jfpp.15146

Kutu, F.R., Magongwa, S.M., 2017. Effect of pre-treatment and preservation methods on the nutritional quality of bitter gourd leaves from two different sources. Res. Crop. 18, 675–682. doi:10.5958/2348-7542.2017.00115.2

Lambein, F., Travella, S., Kuo, Y.H., Van Montagu, M., Heijde, M., 2019. Grass pea (Lathyrus sativus L.): orphan crop, nutraceutical or just plain food? Planta 250, 821–838. doi:10.1007/s00425-018-03084-0

Levy, A., Milo, J., Ashri, A., Palevitch, D., 1983. Heterosis and correlation analysis of the vegetative components and ajmalicine content in the roots of the medicinal plant-Catharanthus roseus (L.) G. Don. Euphytica 32, 557–564. doi:10.1007/BF00021468

Liu, F., Guo, D.D., Tu, Y.H., Xue, Y.R., Gao, Y., Guo, M.L., 2016. Identification of reference genes for gene expression normalization in safflower (Carthamus tinctorius). Rev. Bras. Farmacogn. 26, 564–570. doi:10.1016/j.bjp.2016.05.006

Manners, R., van Etten, J., 2018. Are agricultural researchers working on the right crops to enable food and nutrition security under future climates? Glob. Environ. Chang. 53, 182–194. doi:10.1016/j.gloenvcha.2018.09.010

Masenya, T.A., Pofu, K.M., Mashela, P.W., 2020. Responses of cancer bush (Sutherlandia frutescens) and meloidogyne javanica to increasing concentration of nemafric-bl phytonematicide. Res. Crop. 21, 615–620. doi:10.31830/2348-7542.2020.096

Masondo, N.A., Finnie, J.F., Van Staden, J., 2014. Pharmacological potential and conservation prospect of the genus Eucomis (Hyacinthaceae) endemic to southern Africa. J. Ethnopharmacol. 151, 44–53. doi:10.1016/j.jep.2013.11.002

McCartan, S.A., Van Staden, J., 1999. Micropropagation of members of the Hyacinthaceae with medicinal and ornamental potential - A review. South African J. Bot. 65, 361–369. doi:10.1016/S0254-6299(15)31023-1

Ndiku, H., Mwitari, P., Irungu, B., Ngule, C., 2016. Antibacterial Activity of Pennisetum glaucum Crude Extracts and Pre-purified Fractions against Selected Pathogenic Microorganisms. Br. J. Pharm. Res. 14, 1–9. doi:10.9734/bjpr/2016/28434

Neergheen-Bhujun, V.S., Ruhomally, Z.B., Dunneram, Y., Boojhawon, R., Chan Sun, M., 2020. Consumption patterns, determinants and barriers of the underutilised Moringa oleifera Lam in Mauritius. South African J. Bot. 129, 91–99. doi:10.1016/j.sajb.2019.01.027

Nyadanu, D., Lowor, S.T., 2015. Promoting competitiveness of neglected and underutilized crop species: comparative analysis of nutritional composition of indigenous and exotic leafy and fruit vegetables in Ghana. Genet. Resour. Crop Evol. 62, 131–140. doi:10.1007/s10722-014-0162-x

Obidiegwu, J.E., Lyons, J.B., Chilaka, C.A., 2020. The dioscorea genus (Yam)—An appraisal of nutritional and therapeutic potentials. Foods 9, 1–45. doi:10.3390/foods9091304

Odebunmi, E., Oluwaniyi, O.O., 2010. Comparative proximate analysis of some food condiments Heavy metal adsorption onto nanoparticles View project Heterogeneous Catalytic Hydrodeoxygenation k View project Comparative Proximate Analysis of Some Food Condiments, Article in Journal of Applied Sciences Research.

Ojiem, J.O., Vanlauwe, B., De Ridder, N., Giller, K.E., 2007. Niche-based assessment of contributions of legumes to the nitrogen economy of Western Kenya smallholder farms. Plant Soil 292, 119–135. doi:10.1007/s11104-007-9207-7

Okole, B.N., Odhav, B., 2004. Commercialisation of plants in Africa. South African J. Bot. 70, 109–115. doi:10.1016/S0254-6299(15)30270-2

Olayinka Atoyebi, J., Osilesi, O., Adebawo, O., Abberton, M., 2017. Evaluation of Nutrient Parameters of Selected African Accessions of Bambara Groundnut (Vigna subterranea (L.) Verdc.). Am. J. Food Nutr. 5, 83–89. doi:10.12691/ajfn-5-3-1

Onawola, O., Asagbra, A., 2012. Comparative Soluble Nutrient Value of Ogiri Obtained From Dehulled and Undehulled Boiled Melon Seeds ( Cucumeropsis mannii ) 4, 10–16.

Osei, K., Ennin, S.A., Aighewi, B., Aidoo, A.K., Lamptey, J.N.L., Mochiah, M.B., Aihebhoria, D., Adomako, J., Appiah-Kubi, Z., Mensah, G.O., Asante, B.O., Adu, J.O., Osuman, A.S., 2019. Enhancing productivity of farmer-saved seed yam in Ghana: Positive selection and neem leaf powder factors. African Crop Sci. J. 27, 631. doi:10.4314/acsj.v27i4.6

Ouma, G., Jeruto, P., 2010. Sustainable horticultural crop production through intercropping: The case of fruits and vegetable crops: A review. Agric. Biol. J. North Am. 1, 1098–1105. doi:10.5251/abjna.2010.1.5.1098.1105

Palanisamy, A., Kannan, D., Kavitha, M., Ravindran, C., Rajangam, J., 2019. Potential vegetable crops-need for exploration and conservation for nutritional security for rural livelihoods. Acta Hortic. 1241, 69–74. doi:10.17660/ActaHortic.2019.1241.11

Pande, V.C., Kurothe, R.S., Kumar, G., Singh, H.B., Tiwari, S.P., 2018. Economic assessment of agri-horticulture production systems on reclaimed ravine lands in Western India. Agrofor. Syst. 92, 195–211. doi:10.1007/s10457-016-0025-x

Paul, C., Debnath, A., Chakraborty, K., Ghosh, S., Bhattacharjee, A., Debnath, B., 2020. Sex-specific variations in phytochemicals and antimicrobial potentiality of Dioscorea. Futur. J. Pharm. Sci. 6. doi:10.1186/s43094-020-00076-4

Publishers, W.A., 2018. Diversity and change in food wellbeing. Divers. Chang. food wellbeing 43–69. doi:10.3920/978-90-8686-864-3

Pugalenthi, M., Vadivel, V., Siddhuraju, P., 2005. Alternative food/feed perspectives of an underutilized legume Mucuna pruriens var. utilis - A review. Plant Foods Hum. Nutr. 60, 201–218. doi:10.1007/s11130-005-8620-4

Rani, M., Punia, D., 2015. Nutritional evaluation of matar prepared incorporating green beans powder. Ann. Biol. 31, 161–163.

Rastogi, A., Shukla, S., 2013. Amaranth: A New Millennium Crop of Nutraceutical Values. Crit. Rev. Food Sci. Nutr. 53, 109–125. doi:10.1080/10408398.2010.517876

Rathore, R.S., Nawange, D.D., Solanki, R.S., Singh, R.P., 2012. Identification of suitable ideotypes of blackgram (Vigna Mungo L. Hepper) for intercropping with sorghum (Sorghum Bicolar L. Moench). Legum. Res. 35, 72–74.

Sales, Z.G., Juanico, C.B., Dizon, E.I., Hurtada, W.A., 2018. Indigenous pigmented corn (Zea mays L.) flour as substitute for all-purpose flour to improve the sensory characteristics and nutrient content of crackers. Malays. J. Nutr. 24, 617–625.

Samarakoon, S.M.P.H., Ranil, R.H.G., Fonseka, R.M., Sivarnanthawerl, T., Fonseka, H., Senarathna, S.M.A.C., Sarananda, K.H., Dissanayaka, D., Welegama, H.M.V.T., Niran, H.M.L., 2018. Morphological characterization and assessment of selected biochemical properties of Solatium insanum, L.: Screening of local plant genetic resources for crop improvement. J. Agric. Sci. - Sri Lanka 13, 45–54. doi:10.4038/jas.v13i1.8299

Sangakkara, U.R., Richner, W., Schneider, M.K., Stamp, P., 2003. Impact of intercropping beans (Phaseolus vulgaris L.) and sunhemp (Crotalaria juncea L.) on growth, yields and nitrogen uptake of maize (Zea mays L.) grown in the humid tropics during the minor rainy season. Maydica 48, 233–238.

Sarkar, D., Walker-Swaney, J., Shetty, K., 2020. Food diversity and indigenous food systems to combat diet-linked chronic diseases. Curr. Dev. Nutr. 4, 3–11. doi:10.1093/CDN/NZZ099

Saroop, S., Kaul, V., 2015. Cleome viscosa: a promising underutilized minor crop. Genet. Resour. Crop Evol. 62, 1121–1126. doi:10.1007/s10722-015-0305-8

Sennhenn, A., Njarui, D.M.G.M.G., Maass, B.L.L., Whitbread, A.M.M., 2017. Exploring niches for short-season grain legumes in semi-arid eastern Kenya — Coping with the impacts of climate variability. Front. Plant Sci. 8. doi:10.3389/fpls.2017.00699

Sharifi-Rad, J., Sharifi-Rad, M., Teixeira da Silva, J.A., 2016. Morphological, physiological and biochemical responses of crops (Zea mays L., Phaseolus vulgaris L.), medicinal plants (Hyssopus officinalis L., Nigella sativa L.), and weeds (Amaranthus retroflexus L., Taraxacum officinale F. H. Wigg) exposed to SiO2 nan. J. Agric. Sci. Technol. 18, 1027–1040.

Singh, B., Bhatt, S., Uniyal, P., Pal, V., Chauhan, D.S., 2010. In vitro bioassay to study phytotoxicity effects of Stevia rebaudiana on food crops. J. Trop. For. Sci. 22, 364–369.

Singh, S., Waman, A.A., Bohra, P., Gautam, R.K., Roy, S.D., 2016. Conservation and sustainable utilization of horticultural biodiversity in tropical Andaman and Nicobar Islands, India. Genet. Resour. Crop Evol. 63, 1431–1445. doi:10.1007/s10722-016-0445-5

Slabbert, E.L., Malgas, R.R., Veldtman, R., Addison, P., 2019. Honeybush (Cyclopia spp.) phenology and associated arthropod diversity in the Overberg region, South Africa. Bothalia 49, 1–13. doi:10.4102/abc.v49i1.2430

Soltani, F., Karimi, R., Kashi, A., 2016. Essential oil composition and total phenolic compounds of naked and normal seed types of different accessions of Cucurbita pepo L. in Iran. Iran. J. Plant Physiol. 7, 1889–1897. doi:10.22034/ijpp.2016.532428

Son, H.N., Kingsbury, A., Hoa, H.T., 2021. Indigenous knowledge and the enhancement of community resilience to climate change in the Northern Mountainous Region of Vietnam. Agroecol. Sustain. Food Syst. 45, 499–522. doi:10.1080/21683565.2020.1829777

Srivastava, R., Srivastava, V., Singh, A., 2021. Multipurpose Benefits of an Underexplored Species Purslane (Portulaca oleracea L.): A Critical Review. Environ. Manage. doi:10.1007/s00267-021-01456-z

Sujatha, S., Bhat, R., Kannan, C., Balasimha, D., 2011. Impact of intercropping of medicinal and aromatic plants with organic farming approach on resource use efficiency in arecanut (Areca catechu L.) plantation in India. Ind. Crops Prod. 33, 78–83. doi:10.1016/j.indcrop.2010.09.001

Tchokponhoué, D.A., N’Danikou, S., Hale, I., Van Deynze, A., Achigan-Dako, E.G., 2017. Early fruiting in Synsepalum dulcificum (Schumach. & Thonn.) Daniell juveniles induced by water and inorganic nutrient management. F1000Research 6, 1–19. doi:10.12688/f1000research.11091.1

Tesfay, S.Z., Bertling, I., Odindo, A.O., Seyoum Workneh, T., Mathaba, N., 2011. Levels of anti-oxidants in different parts of moringa (Moringa oleifera) seedling. African J. Agric. Res. 6, 5123–5132. doi:10.5897/AJAR11.1101

Vadivel, V., Doss, A., Pugalenthi, M., 2010. Evaluation of nutritional value and protein quality of raw and differentially processed sword bean [Canavalia gladiata (Jacq.) DC.] seeds. African J. Food, Agric. Nutr. Dev. 10. doi:10.4314/ajfand.v10i7.59034

Van Wyk, B.-E., 2011. The potential of South African plants in the development of new medicinal products. South African J. Bot. 77, 812–829. doi:https://doi.org/10.1016/j.sajb.2011.08.011

van Wyk, B.E., 2008. A broad review of commercially important southern African medicinal plants. J. Ethnopharmacol. 119, 342–355. doi:10.1016/j.jep.2008.05.029

Van Wyk, B.E., Viljoen, A., 2011. Special issue on economic botany. South African J. Bot. 77, 809–811. doi:10.1016/j.sajb.2011.09.008

Veeresh Kumar, Uthappa, A.R., Srivastava, M., Vijay, D., Kumaranag, K.M., Manjunatha, N., Rana, M., Newaj, R., Handa, A.K., Chaturvedi, O.P., Kumar, V., Uthappa, A.R., Srivastava, M., Vijay, D., Kumaranag, K.M., Manjunatha, N., Rana, M., Newaj, R., Handa, A.K., Chaturvedi, O.P., Veeresh Kumar, Uthappa, A.R., Srivastava, M., Vijay, D., Kumaranag, K.M., Manjunatha, N., Rana, M., Newaj, R., Handa, A.K., Chaturvedi, O.P., 2017. Floral biology of Grewia flavescens Juss.: an underutilized crop. Genet. Resour. Crop Evol. 64, 1789–1795. doi:10.1007/s10722-017-0536-y

Velasco, P., Cartea, M.E., González, C., Vilar, M., Ordás, A., 2007. Factors affecting the glucosinolate content of kale (Brassica oleracea acephala Group). J. Agric. Food Chem. 55, 955–962. doi:10.1021/jf0624897

Venter, S.L., Fouché, H.J., De Wit, M., Mavengahama, S., Coetzer, G.M., Swart, W.J., Amonsou, E.O., 2019. The effect of fostering partnerships on broadening the food base: The role of cactus pear, an underutilised crop with unlimited potential – the South African perspective. Acta Hortic. 1247, 237–244. doi:10.17660/ActaHortic.2019.1247.32

Verma, A., Srivastava, K., Singh, S., Singh, H., 2017. Nutraceuticals and innovative food products for healthy living and preventive care i, 304–322.

Xavier, F., Kumar, R., Yadav, R.K., Behera, T.K., Khade, Y.P., 2019. Studies on combining ability of okra genotypes for protein, total dietary fibre and mineral content. Indian J. Hortic. 76, 672–677. doi:10.5958/0974-0112.2019.00107.5

Xego, S., Kambizi, L., Nchu, F., 2016. Threatened medicinal plants of South Africa: Case of the family hyacinthaceae. African J. Tradit. Complement. Altern. Med. 13, 169–180. doi:10.4314/ajtcam.v13i3.20

Zhang, D.Y., Wan, Y., Hao, J.Y., Hu, R.Z., Chen, C., Yao, X.H., Zhao, W.G., Liu, Z.Y., Li, L., 2018. Evaluation of the alkaloid, polyphenols, and antioxidant contents of various mulberry cultivars from different planting areas in eastern China. Ind. Crops Prod. 122, 298–307. doi:10.1016/j.indcrop.2018.05.065

Zhang, H., Tan, J., Zhang, M., Huang, S., Chen, X., 2020. Comparative transcriptomic analysis of two bottle gourd accessions differing in fruit size. Genes (Basel). 11. doi:10.3390/genes11040359

Zonyane, S., Chen, L., Xu, M.J., Gong, Z.N., Xu, S., Makunga, N.P., 2019. Geographic-based metabolomic variation and toxicity analysis of Sutherlandia frutescens L. R.Br. – An emerging medicinal crop in South Africa. Ind. Crops Prod. 133, 414–423. doi:10.1016/j.indcrop.2019.03.010

Zubair, M., Nybom, H., Lindholm, C., Rumpunen, K., 2011. Major polyphenols in aerial organs of greater plantain (Plantago major L.), and effects of drying temperature on polyphenol contents in the leaves. Sci. Hortic. (Amsterdam). 128, 523–529. doi:10.1016/j.scienta.2011.03.001
